# *Gasterophilus flavipes* (Oestridae: Gasterophilinae): A horse stomach bot fly brought back from oblivion with morphological and molecular evidence

**DOI:** 10.1371/journal.pone.0220820

**Published:** 2019-08-12

**Authors:** Xin-yu Li, Thomas Pape, Dong Zhang

**Affiliations:** 1 Key Laboratory of Non-Invasive Research Technology for Endangered Species, School of Nature Conservation, Beijing Forestry University, Beijing, China; 2 Natural History Museum of Denmark, University of Copenhagen, Copenhagen, Denmark; University of Lincoln, UNITED KINGDOM

## Abstract

Species of *Gasterophilus* Leach are obligate parasites in domestic and wild equids and responsible for cosmopolitan gasterophilosis. Although with only eight species known so far, they have received considerable attention because of their significant veterinary and economic importance. Surprisingly, we found that *G*. *flavipes* (Olivier) is a valid species based on morphological characters from male, female and the egg, after spending half a century as a synonym of *G*. *haemorrhoidalis* (Linnaeus). In the present study, *G*. *flavipes*, *G*. *haemorrhoidalis* and *G*. *inermis* (Brauer), which are the three closely related species possessing a remarkable mixture of shared morphological characters, are diagnosed and comparatively redescribed; the key to separate adults and eggs are provided, together with a series of high-resolution photographs from all the body parts. COI barcodes do not allow for a separation of *G*. *flavipes*, *G*. *haemorrhoidalis* and *G*. *inermis*, but showed a closer relationship between *G*. *flavipes* and *G*. *haemorrhoidalis* than the other two combinations, which is consistent with the morphological evidence. Geographically, *G*. *flavipes* seems to be common and widespread in the warmer parts of the Palaearctic region. Thus, the epidemiology of gasterophilosis where *G*. *flavipes* is known or supposed to occur calls for a more careful veterinarian re-assessment. A decline in the populations of *Gasterophilus* spp. has been noticed in Europe, but all seven Palaearctic species of *Gasterophilus* appear to maintain stable populations in Xinjiang (China), which may be explained by a higher biodiversity of equids and less use of anti-parasitic treatments in Xinjiang than in Europe. Our study shows that morphological characters still provide the solid backbone in classification of *Gasterophilus* at species-level, and updated diagnoses and a key is provided to distinguish *G*. *flavipes*, *G*. *haemorrhoidalis* and *G*. *inermis*, and to facilitate studies of epidemiology, phylogeny and host-parasite co-evolution.

## Introduction

Species of *Gasterophilus* Leach (Oestridae: Gasterphilinae) are obligate parasites adapted to a larval life in the intestinal tract of equids (including horses, donkeys, asses and zebras) [1–6]. Although only nine species are known (present study), the genus has become near cosmopolitan by the association with domestic hosts [3,4,6–9]. *Gasterophilus* species are responsible for gasterophilosis in domestic and wild equids, which can lead to serious injuries (e.g. destruction of the gums, ulcerations, peritonitis, anemia, heavy debilitation and blockage of the gastrointestinal tract), or even death to the hosts [10–14].

Due to the significant veterinary and economic importance of *Gasterophilus* species, they have received considerable attention regarding identification, distribution, infectious intensity and incidence [15–18]. It has been suggested that species of *Gasterophilus* may be threatened with extinction because of increased and widespread use of broad spectrum antiparasitics (e.g. macrocyclic lactones, which provides exquisite sensitivity to *Gasterophilus*) [15,19] and a decreased number of other equids (e.g. donkeys). Regional decline of biodiversity and risks of extinction of *Gasterophilus* species have already been noticed in Italy [15].

Interestingly, all the Palaearctic *Gasterophilus* species have been recently reported in Xinjiang Autonomous Region, China, where they appear to maintain large and stable populations [17,20,21]. Surprisingly, numerous males and females of *Gasterophilus* were collected during recent epidemiological investigations in Kalamaili, Xinjiang, which could not be identified using the keys from Grunin [3] and Zumpt [4]. These specimens possess a remarkable mixture of features shared with *G*. *haemorrhoidalis* (Linnaeus) and *G*. *inermis* (Brauer), but distinct differences in setal colouration and shape of male terminalia strongly suggest that these specimens represent a valid species. After a thorough examination of morphological characters and available literature, we found that habitus, colour pattern and male terminalia of this species match those of *G*. *flavipes* [22], whose male terminalia were thoroughly documented by Patton [23]. The name *G*. *flavipes* was subsequently synonymised by Grunin [3] and Zumpt and Paterson [24] under *G*. *haemorrhoidalis*.

Thus, the aim of this study is to resurrect *G*. *flavipes* as a valid species utilizing both morphological and molecular data, including the first description of the female and the egg, present a reliable key to separate adult flies of the three morphologically similar species, *G*. *flavipes*, *G*. *haemorrhoidalis* and *G*. *inermis*, and provide morphological, molecular and distribution data to facilitate epidemiological and host-parasite investigations of *Gasterophilus*.

## Material and methods

### Ethics statement

This study was carried out under a bio-specimen collecting permit issued by the current authority (Hong-jun Chu) of Forestry Bureau, Xinjiang. There is no number for the issued permit.

### Sample collection

*Gasterophius flavipes* adults (11 males, 15 females) were sorted out from a single sample of a Malaise trap deployed for 10 days at the Qiaomuxibai water reservoir (45°13’48”N; 89°03’00”E), Kalamaili, Xinjiang, China, in 2017. The specimens are deposited at Beijing Forestry University, Beijing, China, and Natural History Museum of Denmark, University of Copenhagen, Denmark.

### Morphological and distribution data

Series of photographs were taken using a Visionary Digital Imaging System, with a Canon EOS 7D (Canon, Inc., Tokyo, Japan) and stacked using Zerene Stacker software, or using an Olympus SZX16 stereoscopic microscope (Olympus Corp., Tokyo, Japan) equipped with a Canon 600D digital camera (Canon, Inc., Tokyo, Japan) and stacked using Combine ZP (by Alan Hadley). Superimposed photographs were composed using Adobe Photoshop CS6 (Adobe Systems, Inc., San Jose, CA, U.S.A.) on a Windows 10 platform. Male terminalia were treated in 10% KOH solution before being dissected and the eggs of *G*. *flavipes* were obtained by dissecting a female specimen. Terminology of adult morphology follows Pape [25] and Cumming and Wood [26].

Redescriptions are provided for adults and eggs only. Thorough and comprehensive morphological characters and identification keys to third instar *Gasterophilus* larvae have been updated by Li et al [21], and the third instar of *G*. *flavipes* is still unknown.

Distribution and hosts are given based on information obtained from specimens examined for the present study together with data from Brauer [2], Zumpt [4], Guimarães [27], Guimarães and Papavero [28], Grunin [3], Grunin [29], Pont [7], James [30], Pont [31], Kaboret et al [32], Soós and Minář [8], Pearse et al [33], Xue and Wang [34], Tavassoli and Bakht [35], Mashayekhi and Ashtar [36], de Jong et al [37], and Huang et al [16]. As *G*. *flavipes* is resurrected in the present paper we have given non-vouchered literature records for this species with a question mark.

### DNA extraction, PCR amplification, and sequencing

Three fresh adult specimens (2 males, 1 female) of *G*. *flavipes* were selected for molecular analysis (S1 Table). A small sample of thoracic muscle tissue was dissected from each individual to extract the total genomic DNA using the DNeasy Blood and Tissue kit protocol (Qiagen, Dusseldorf, Germany). The remaining body parts were retained as vouchers and deposited in the entomological collection of Beijing Forestry University. An approximately 650-bp region near the 5’-terminus of the COI, the barcode region (Hebert et al., 2003; hereafter COI-5’), and a 663-bp region near the 3’-terminus of the COI (hereafter COI-3’) were PCR-amplified using the universal barcode primers LCO1490-L (5’-GGTCWACWAATCATAAAGATATTGG-3’) and HCO2198-L (5’-RAAACTTCWGGRTGWCCAAARAATCA-3’) [38,39], and COI-II-F (5’-CCACATTTATTTTGATTTTTTGG-3’) and COI-II-R (5’-TCCAATGCACTAATCTGCCATATTA-3’) [40] according to the *Gasterophilus* DNA barcoding system [21]. PCRs were conducted according to Zhang et al [41], and amplification followed Kutty et al [42]. All PCR products were purified and sequenced both forward and reverse by BGI Inc., Beijing, China. A total of 52 COI sequences obtained from GenBank, and six from Otranto et al [15] (the partial data were published without being uploaded to GenBank), representing all the seven Palaearctic *Gasterophilus* species, are included in our analyses. *Hypoderma lineatum* (Villers) (Hypodermatinae) and *Dermatobia hominis* (Linnaeus, Jr.) (Cuterebrinae) were chosen as outgroups, with the rooting done by *D*. *hominis* (S1 Table).

### DNA sequence editing, assembling and alignment

SeqMan Pro v. 7.1.0 (DNASTAR Inc., USA) was used to edit and assemble the forward and reverse sequences. Alignment was conducted using the online version of MAFFT v. 7 [43] (available at https://mafft.cbrc.jp/alignment/server/), with the algorithm G-INS-i and default parameters. All the sequences are assembled, aligned and available in GenBank under the accession numbers: MK412087–MK412089 (S1 Table).

### DNA sequence analysis

Nucleotide sequence divergences were calculated using Kimura 2-parameter (K2P) model under MEGA X software [44,45]. A neighbour-joining (NJ) analysis under 1,000 bootstrap replicates was conducted in MEGA X to estimate the genetic divergence of COI between specimens [46].

## Results

### Intra- and interspecific divergences of the COI gene among *G*. *flavipes*, *G*. *haemorrhoidalis* and *G*. *inermis*

**Fig 1:**
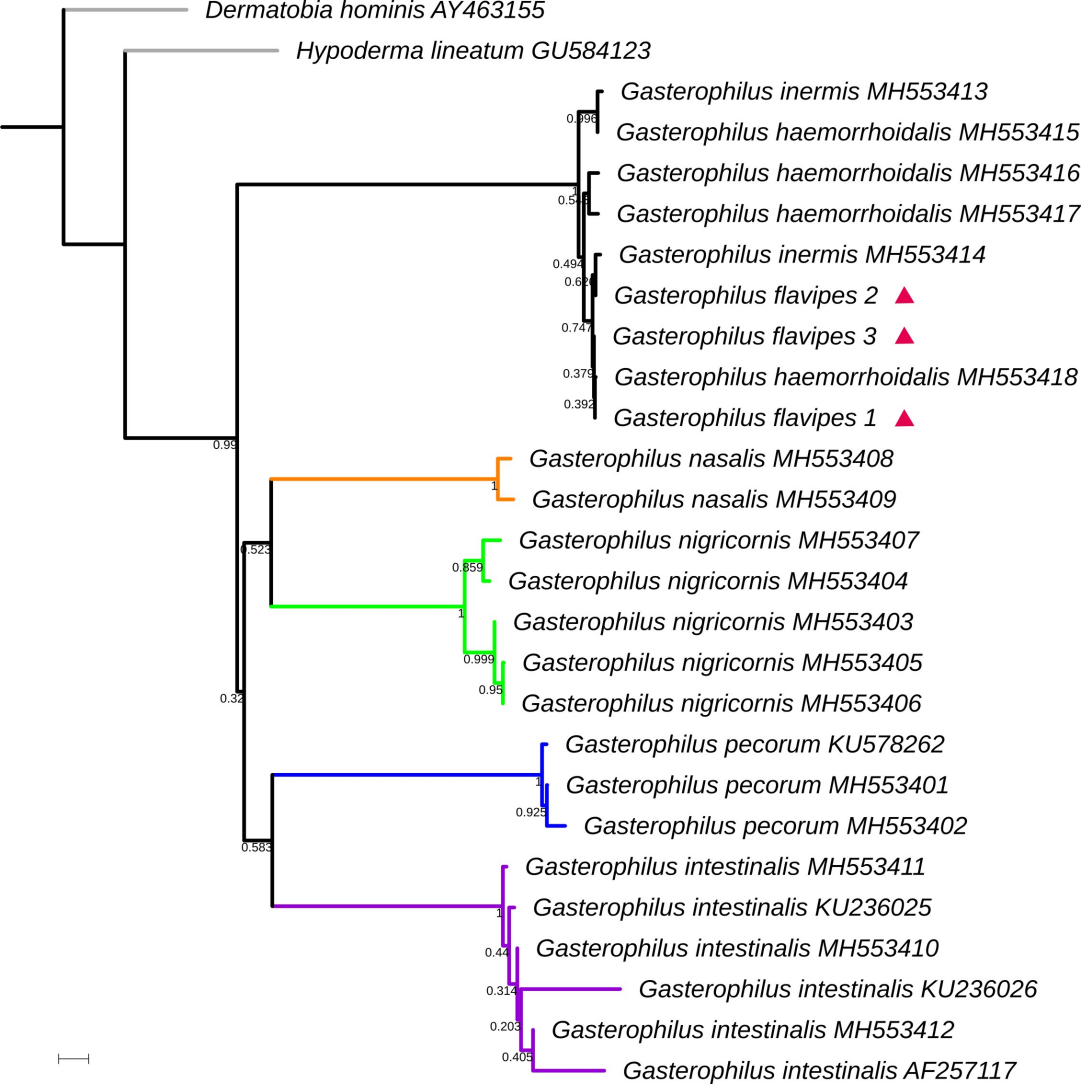
Neighbour-joining (NJ) tree of Kimura-two-parameter (K2P) distances of the traditional DNA barcode region (about 650 bp region near the 5′ terminus of the cytochrome oxidase subunit I) in seven Palaearctic *Gasterophilus* species. Numbers given at branches refer to bootstrap proportions among 1000 bootstrap replicates. Red triangles indicate *G*. *flavipes*. Scale bar represents 0.01 nucleotide substitutions per site.

**Fig 2:**
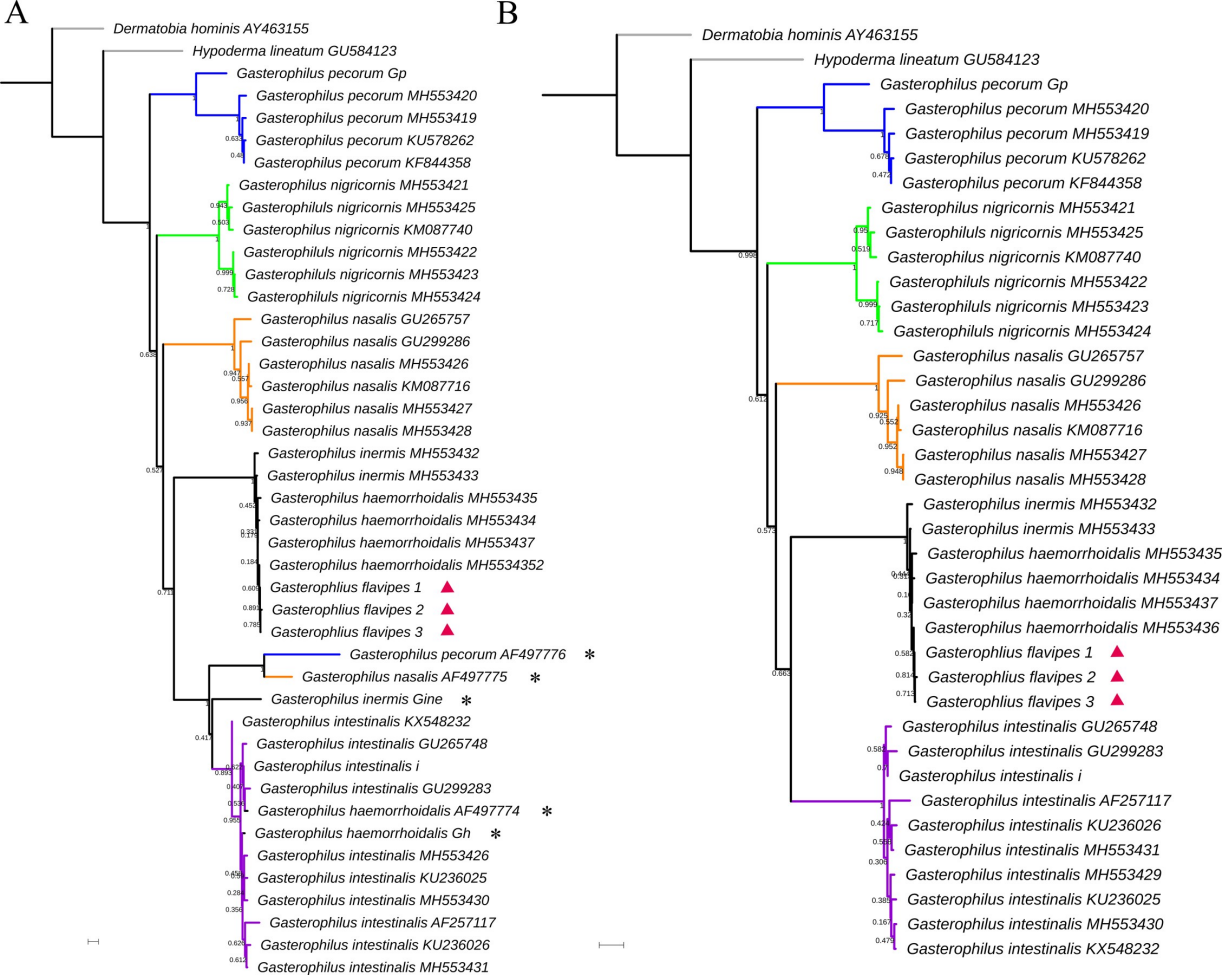
Neighbour-joining (NJ) tree of Kimura-two-parameter distances of a 663 bp region near the 3′ terminus of the cytochrome c oxidase subunit I (COI) (A = all data, B = only verified identifications) in seven Palaearctic *Gasterophilus* species. Numbers given at branches refer to bootstrap proportions among 1000 bootstrap replicates. Red triangles indicate *G*. *flavipes*. Asterisks indicate questionable identifications. Scale bar represents 0.01 nucleotide substitutions per site.

Molecular analysis revealed very low interspecific nucleotide variation of the COI gene for the samples from *G*. *flavipes*, *G*. *haemorrhoidalis* and *G*. *inermis*, which is insufficient for identification and differentiation (Figs 1 and 2). For these three species, intraspecific variation of the COI-5’ barcode region was 0.10% (*G*. *flavipes*, SE = 0.10%), 1.10% (*G*. *haemorrhoidalis*, SE = 0.26%) and 1.42% (*G*. *inermis*, SE = 0.45%) (S2 Table), and for COI-3’ 0.10% (*G*. *flavipes*, SE = 0.10%), 0.30% (*G*. *haemorrhoidalis*, SE = 0.15%) and 0.76% (*G*. *inermis*, SE = 0.32%) (S3 Table). Interspecific variation for COI-5’ranged from 0.67% (*G*. *flavipes* and *G*. *haemorrhoidalis*, SE = 0.22%) to 1.11% (*G*. *haemorrhoidalis* and *G*. *inermis*, SE = 0.25%) (S4 Table); and for COI-3’ from 0.28% (*G*. *flavipes* and *G*. *haemorrhoidalis*, SE = 0.14%) to 0.45% (*G*. *haemorrhoidalis* and *G*. *inermis*, SE = 0.17%) (S5 Table).

### Key to the imagines of *G*. *flavipes*, *G*. *haemorrhoidalis* and *G*. *inermis*

(Figs 3–11 presenting characters in the key)

**Fig 3:**
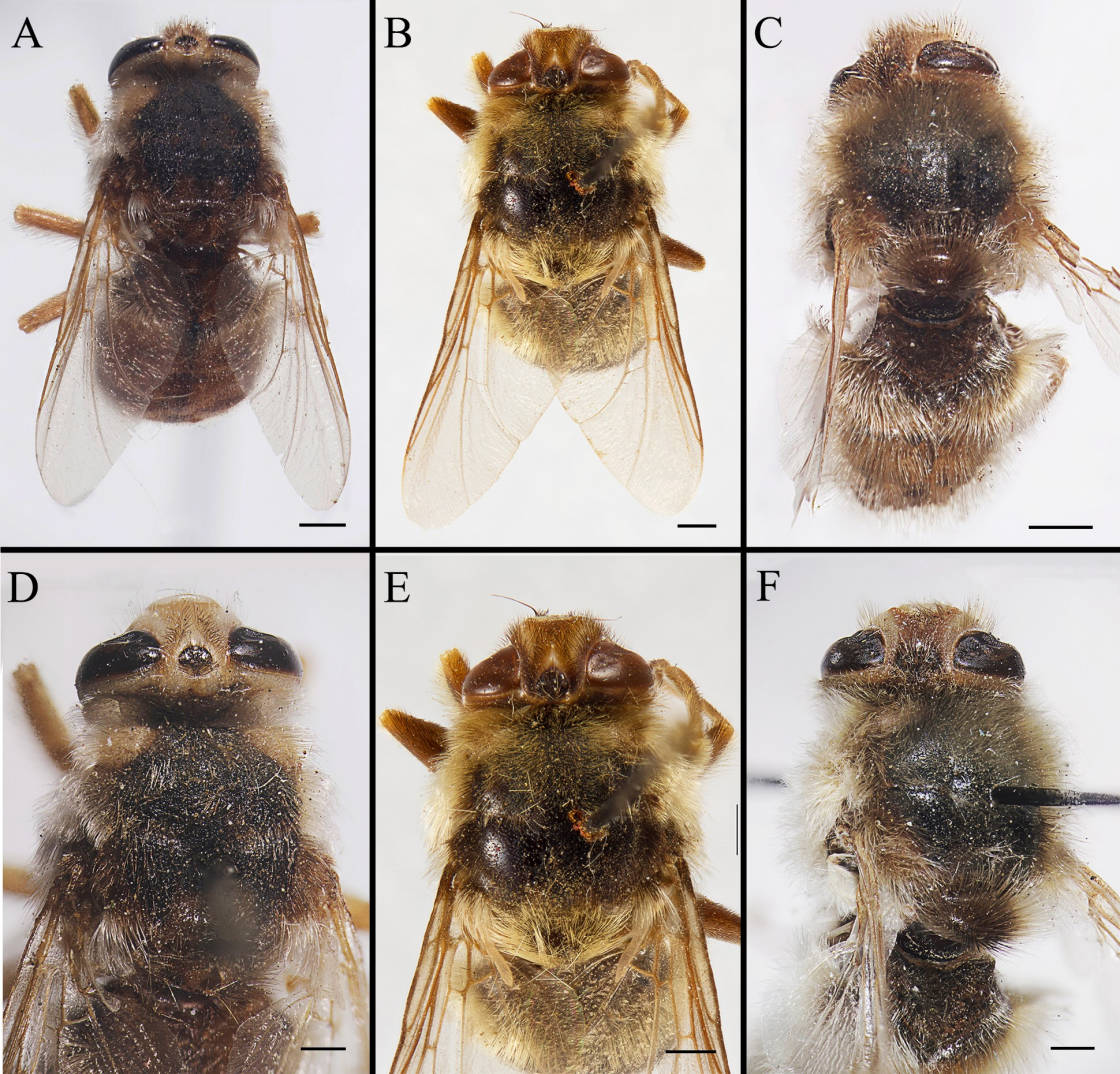
***Gasterophilus* spp., male habitus (A–C) and head and thorax (D–F), dorsal view. A, D.** *G*. *flavipes* (Olivier). B, E. *G*. *haemorrhoidalis* (Linnaeus). C, F. *G*. *inermis* (Brauer). Scale bars: A–F = 0.5 mm.

**Fig 4:**
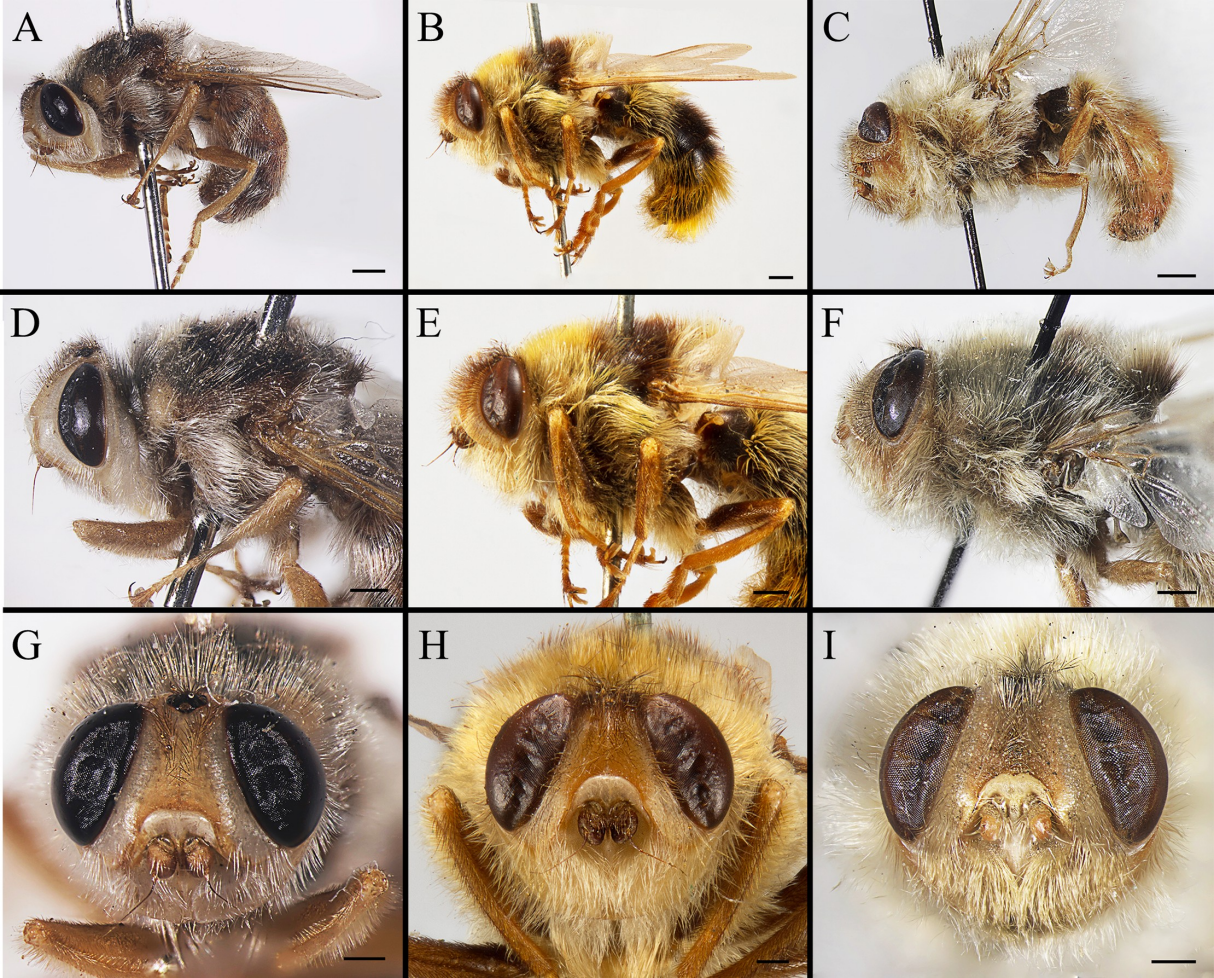
***Gasterophilus* spp., male lateral habitus (A–C), head and thorax in lateral view (D–F) and head in frontal view (G–I).** A, D, G. *G*. *flavipes* (Olivier). B, E, H. *G*. *haemorrhoidalis* (Linnaeus). C, F, I. *G*. *inermis* (Brauer). Scale bars: A–F = 1 mm; G–I = 0.5 mm.

**Fig 5:**
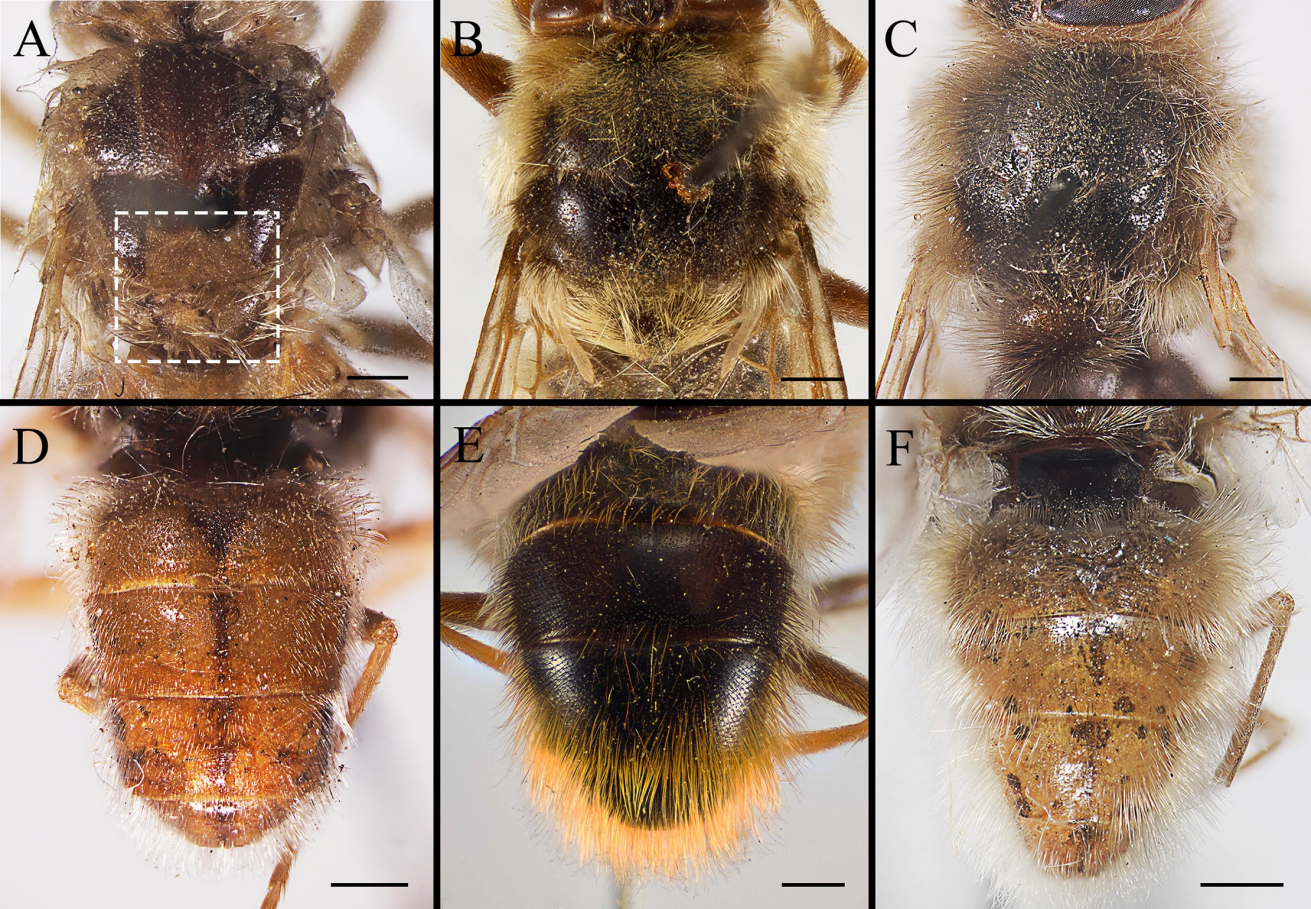
***Gasterophilus* spp., male thorax (A–C) and abdomen (D–F) in dorsal view.** A, D. *G*. *flavipes* (Olivier). B, E. *G*. *haemorrhoidalis* (Linnaeus). C, F. *G*. *inermis* (Brauer). Scale bars: A–F = 1 mm.

**Fig 6:**
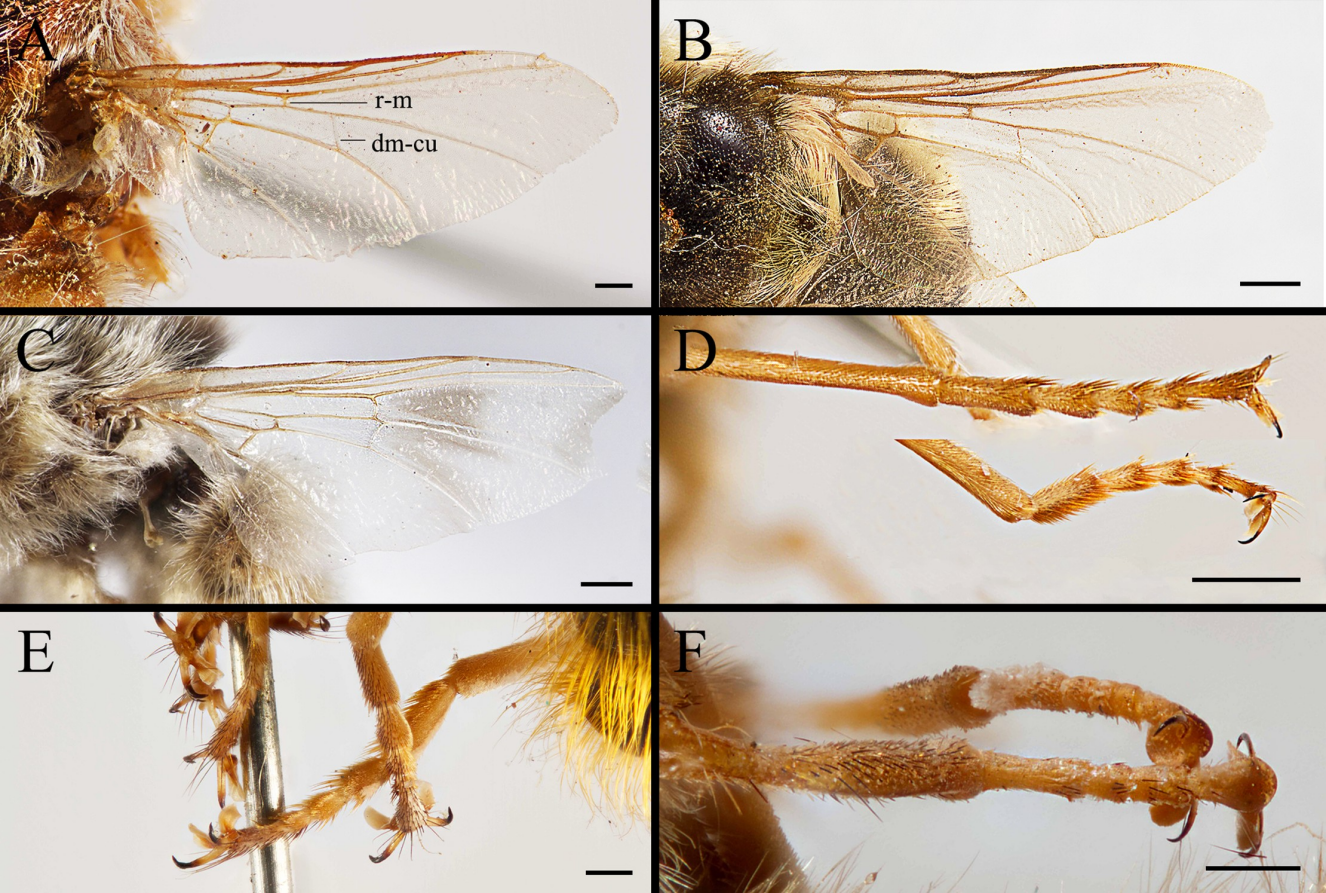
*Gasterophilus* spp., male wing (A–C) and hind legs (D–F). A, D. *G*. *flavipes* (Olivier). B, E. *G*. *haemorrhoidalis* (Linnaeus). C, F. *G*. *inermis* (Brauer). Scale bars: A–F = 0.5 mm.

**Fig 7:**
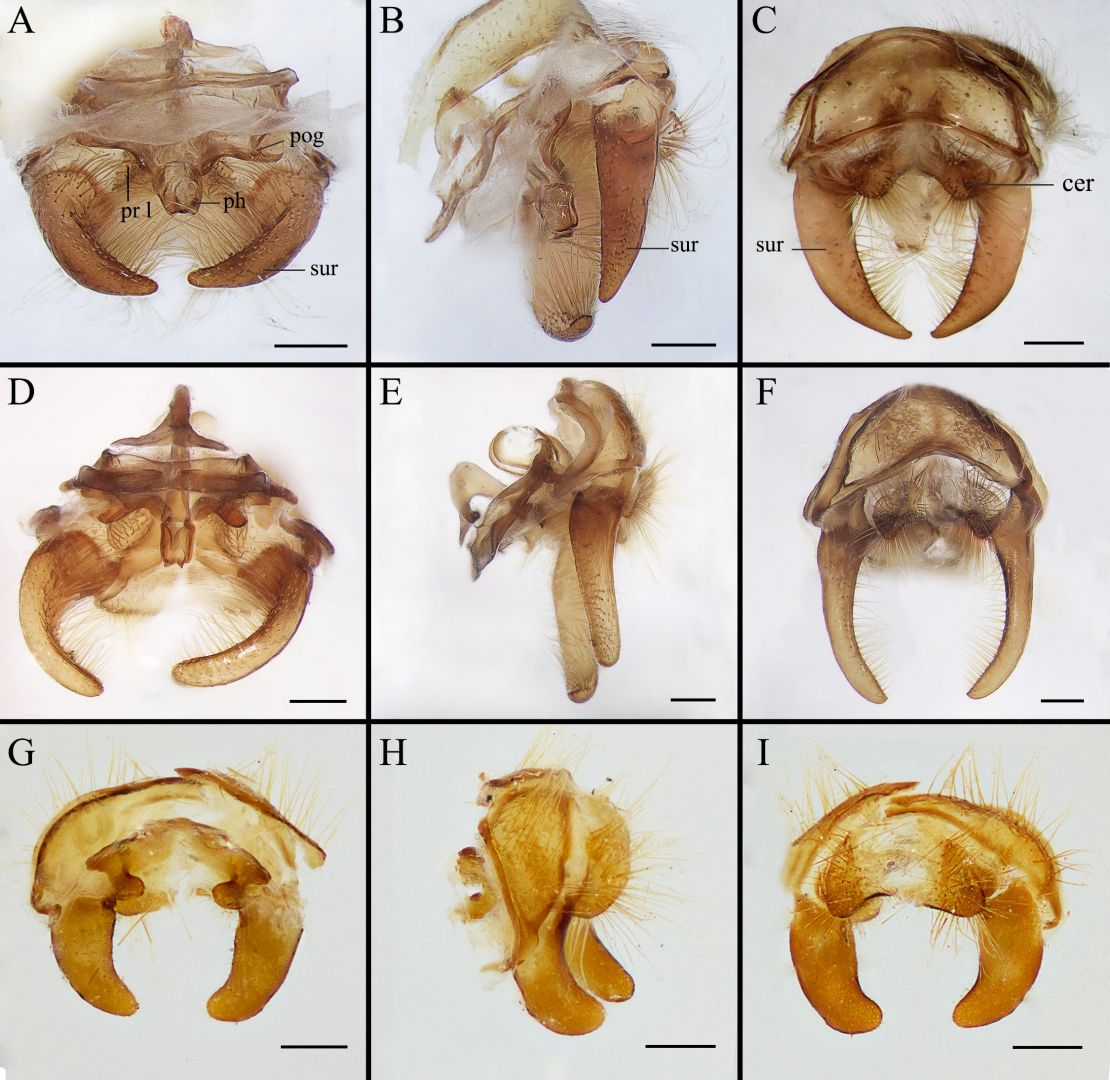
*Gasterophilus* spp., male terminalia. (A, D, G. in anterior view. B, E. H. in left lateral view (mirrored for matching the left view of habitus and head); C, F, I. in dorsal view.). A–C. *G*. *flavipes* (Olivier). D–F. *G*. *haemorrhoidalis* (Linnaeus). G–I. *G*. *inermis* (Brauer). Scale bars: A–I = 0.5 mm. Abbreviations: cer, cercus; ph, phallus; pog, postgonite; pr l, processi longi; sur, surstylus.

**Fig 8:**
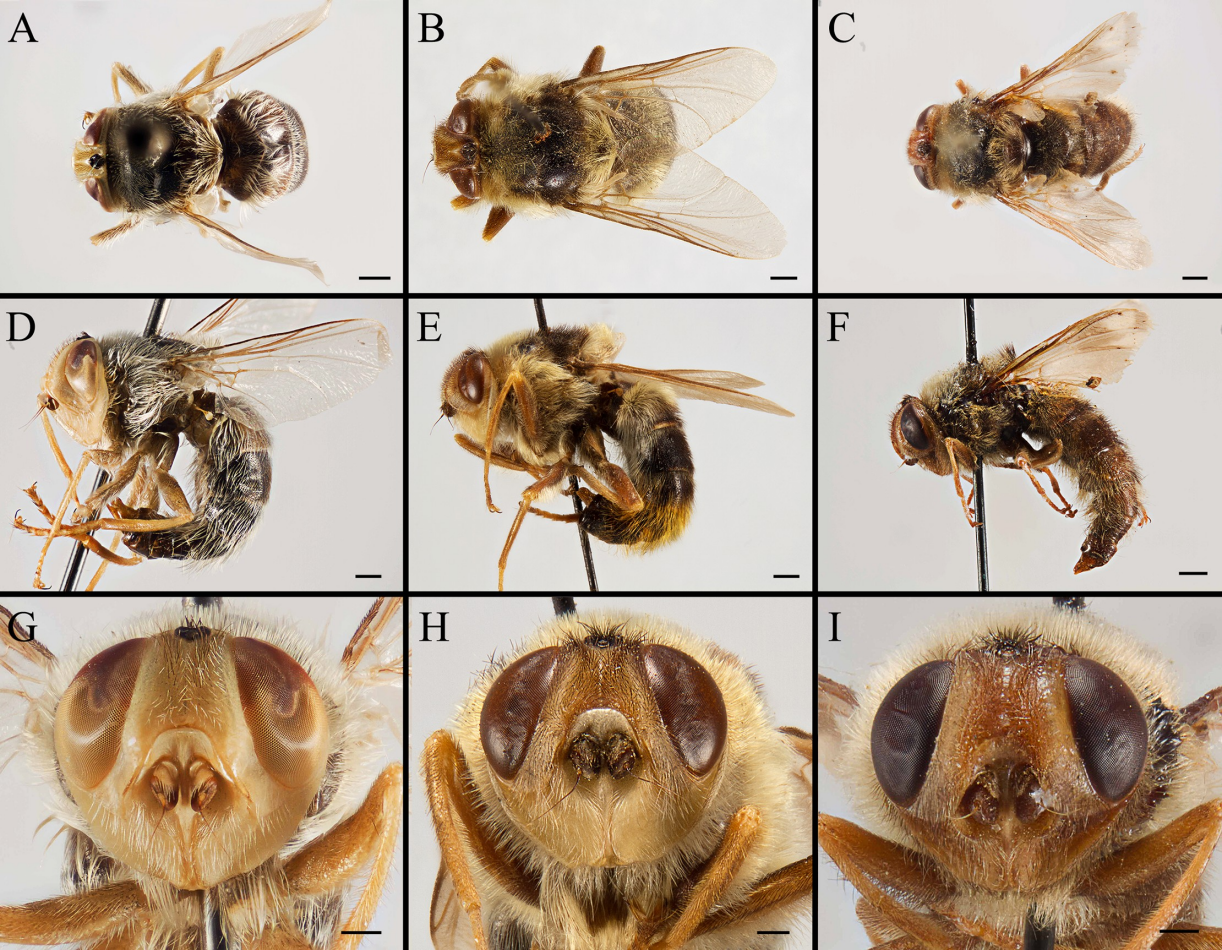
***Gasterophilus* spp., female dorsal (A–C) and lateral habitus (D–F), and head in frontal view (G–I).** A, D, G. *G*. *flavipes* (Olivier). B, E, H. *G*. *haemorrhoidalis* (Linnaeus). C, F, I. *G*. *inermis* (Brauer). Scale bars: A–F = 1mm; D, G–I = 0.5 mm.

**Fig 9:**
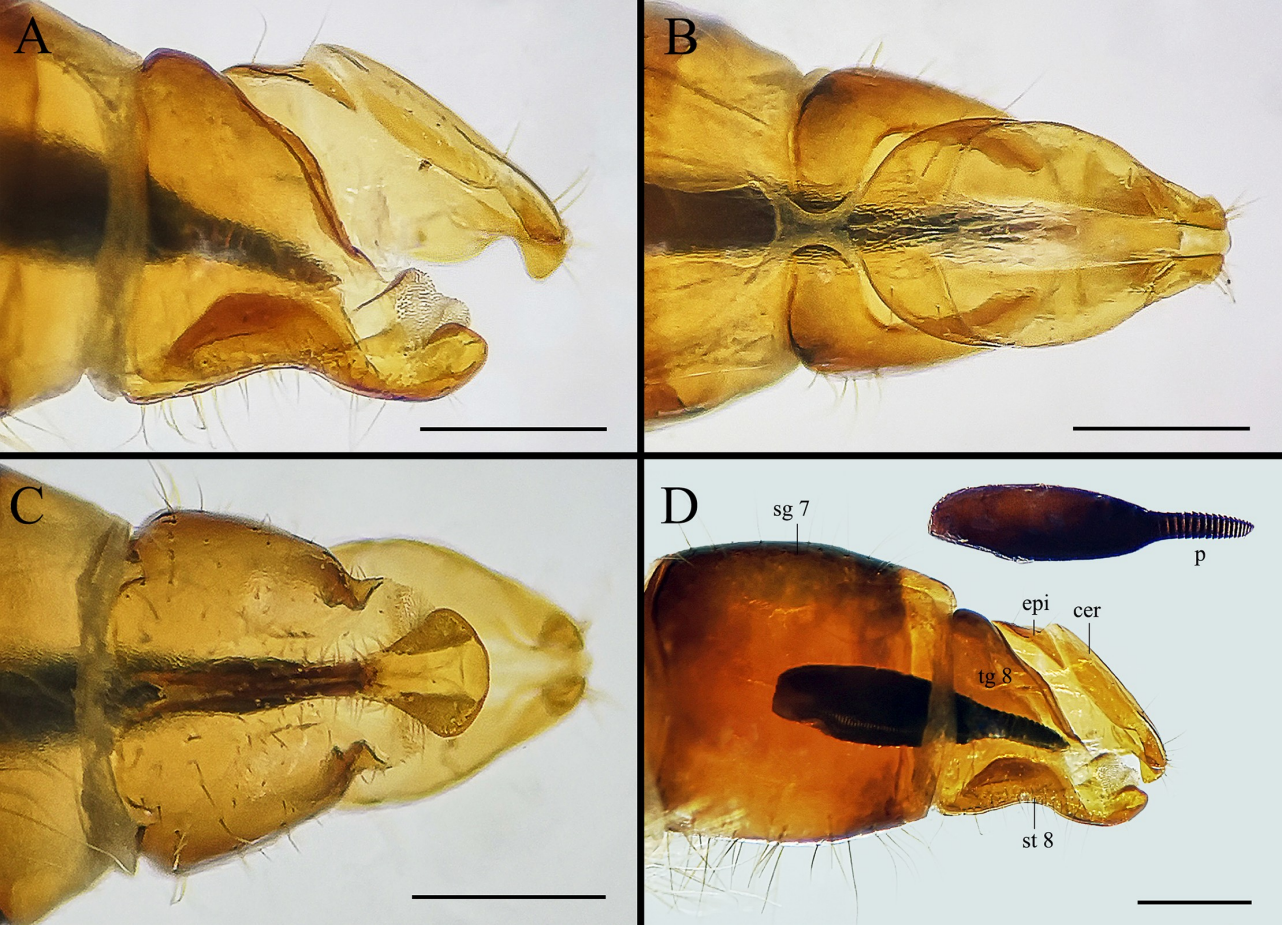
***Gasterophilus flavipes* (Olivier), female terminalia (A–D) and egg (D).** A. Left lateral view. B. Dorsal view. C. Ventral view. D. Left lateral view of the egg, female abdominal segment 7 and genitalia (mirrored for better comparison with 7A). Scale bars: A–D = 0.5 mm. Abbreviations: cer, cercus; epi, epiproct; sg 7, segment 7; p, pedicel; st 8, sternite 8; tg 8, tergite 8.

**Fig 10:**
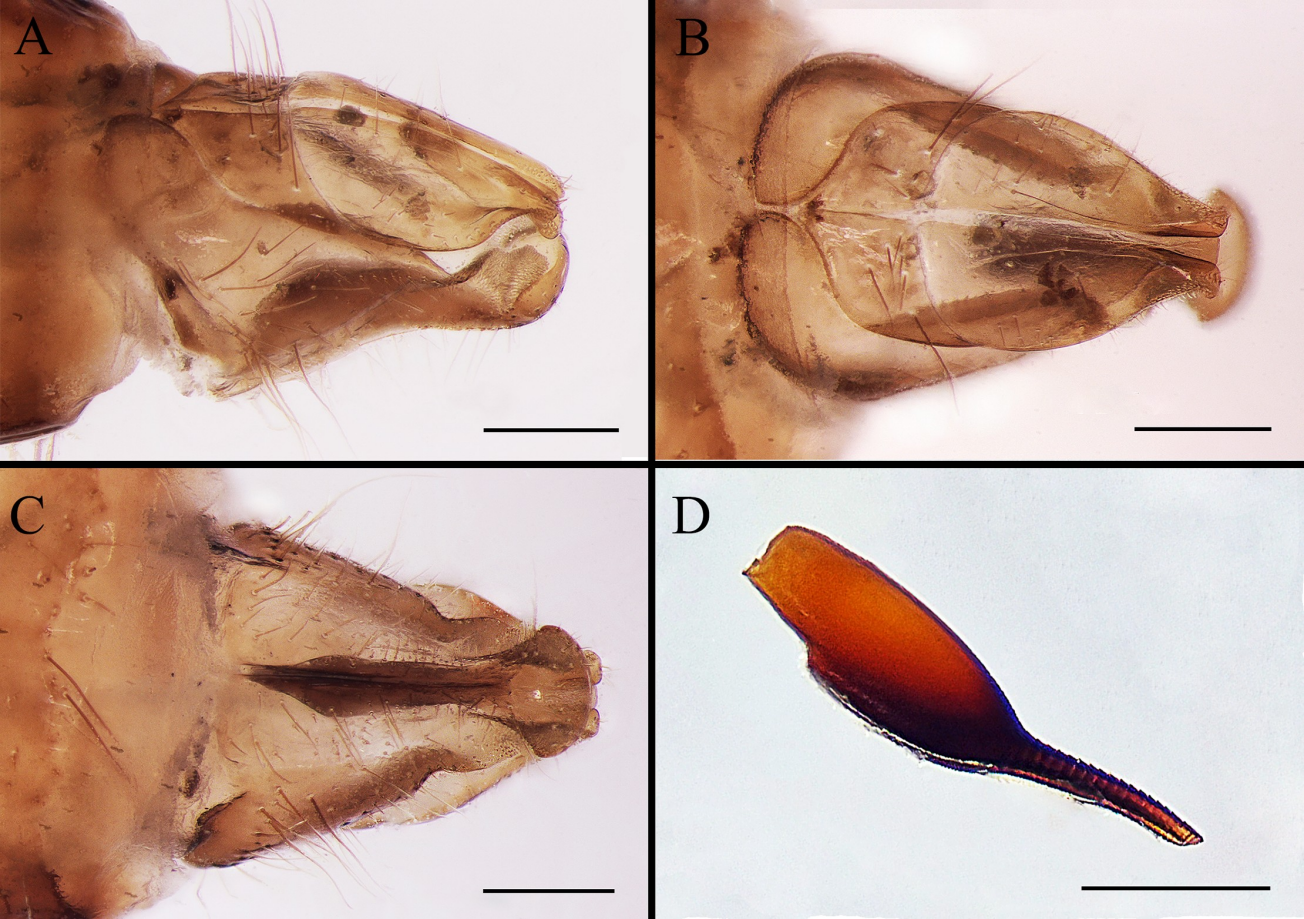
***Gasterophilus haemorrhoidalis* (Linnaeus), female genitalia (A–C) and egg (D).** A. Left lateral view. B. Dorsal view. C. Ventral view. D. Left lateral view of the egg. Scale bars: A–D = 0. 5 mm.

**Fig 11:**
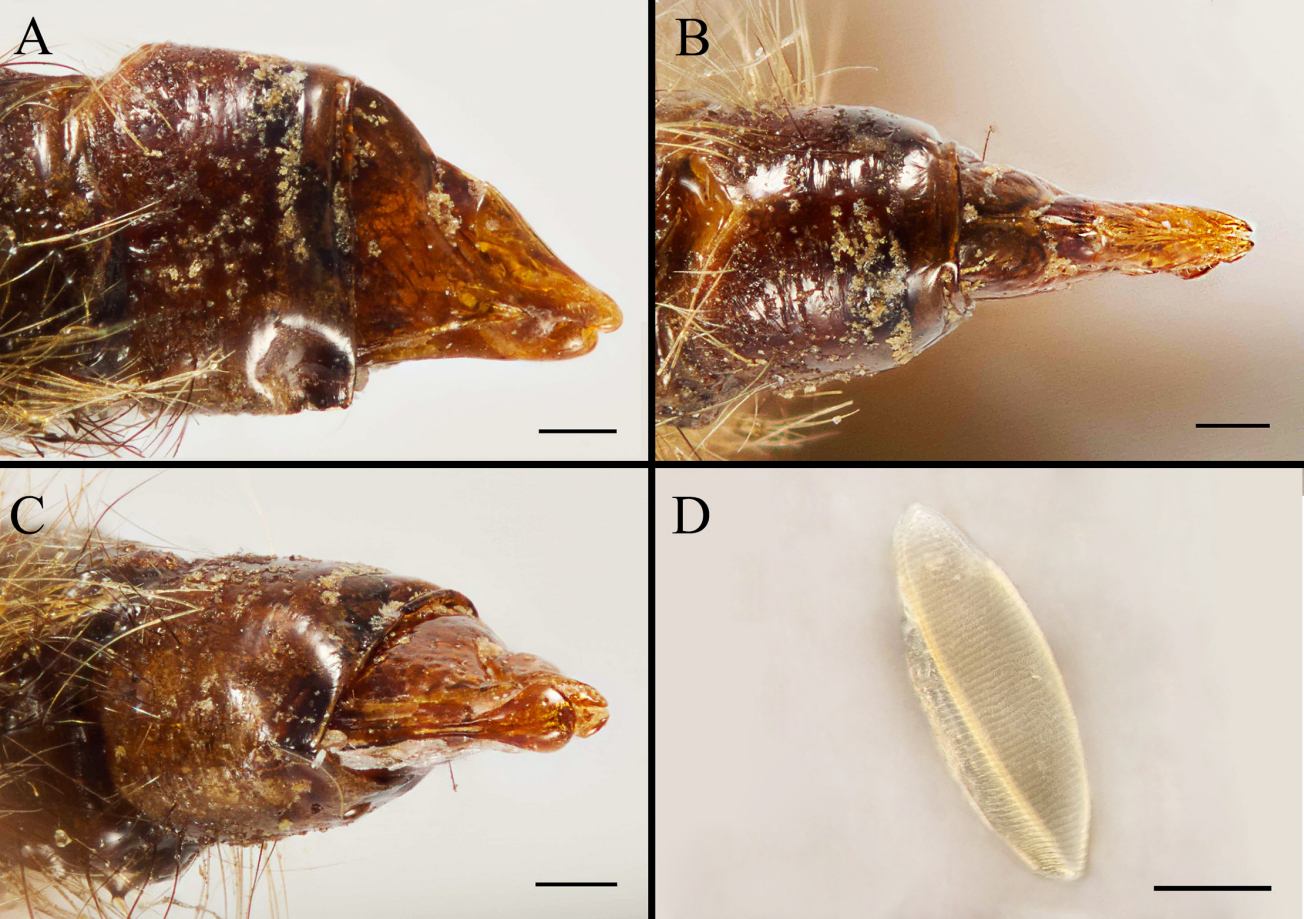
***Gasterophilus inermis* (Brauer), female genitalia (A–C) and egg (D).** A. Left lateral view. B. Dorsal view. C. Ventral view. D. Right lateral view of the egg. Scale bars: A–D = 0. 25 mm.

1. Frontal vitta and fronto-orbital plate ground colour uniformly yellow or dark brown in dorsal view (i.e., without clear colour difference/boundary); length ratio of male cercus and surstylus more than 2/3, male processi longi elongated (Grunin [3]: figs 34–36, 58–60, 68–70; Patton [23]: figs 4, 6); female cercus gradually tapered (Grunin [3]: figs 37–39; Patton [23]: figs 5, 7) …………………………………………Consult the keys of Grunin [3] and Zumpt [4]

--Ground colour of frontal vitta distinctly darker than fronto-orbital plate in dorsal view (i.e., with clear colour difference/boundary) (Fig 3D, 3E and 3F); length ratio of male cercus and surstylus equal or less than 1/3 (Fig 7C, 7F and 7I), male processi longi tubercular (Fig 7A, 7D and 7G); female cercus apex abruptly narrowed (Figs 9B, 10B and 11B) ……………………………………………………………………………………………………………………………………………………2

2. Wing with darkened patches with ill-defined edges, distance between crossvein r-m and dm-cu less than length of r-m (Figs 6C; 8C and 8F) ……………………………………………………………………………………………*Gasterophilus inermis*

--Wing entirely hyaline, distance between crossvein r-m and dm-cu at least twice as long as r-m (Figs 3A and 3B; 6A and 6B; 8A, 8B, 8D and 8E) ……………………………………………………………………………………………………………………………………3

3. Postsutural scutum with a light (yellowish), rectangular area near scutoscutellar suture (Fig 5A); legs yellow; abdominal ground colour yellow, covered with yellow setae (Fig 5D); male with surstylus gradually tapered proximally and distally (Fig 7A–7C); surstylar setae long, reaching the sagittal plane (Fig 7C) …………………………………………………………………………………………………………………………*Gasterophilus flavipes*

--Postsutural scutum with ground colour uniformly brown or black (Fig 5B); legs yellowish brown, with femora distinctly darkened; abdominal ground colour dark brown or black, with reddish-yellow or orangish setae posteriorly (Fig 5E); male with surstylus abruptly swollen at base (Fig 7D–7F); surstylar setae short, reaching at most halfway to the sagittal plane (Fig 7F) …………………………………………………………………………*Gasterophilus haemorrhoidalis*

### Comparative redescription, hosts and distribution of *G*. *flavipes*, *G*. *haemorrhoidalis* and *G*. *inermis*

#### Head

Width slightly less than width of thorax at middle (Fig 3). Head profile oval with largest width at level of antennal insertion (Fig 4). Width of frons 1/4 width of head in dorsal view (Fig 4G–4I). Ocellar triangle dark brown, with dense, dark brown hair-like setae. Each ocellus circled by a dark brown cuticular band. Frontal vitta dark yellow in *G*. *flavipes* and *G*. *inermis*, brown in *G*. *haemorrhoidalis*, with long, dark brown hair-like setae in *G*. *flavipes* and *G*. *haemorrhoidalis*, a mixture of brown and pale hair-like setae in *G*. *inermis*. Fronto-orbital plate pale yellow in *G*. *flavipes* and *G*. *inermis*, brown in *G*. *haemorrhoidalis*, with pale yellow hair-like setae, sparse in *G*. *flavipes*, dense in *G*. *inermis*; while the setae are dark brown in *G*. *haemorrhoidalis*. Antenna inserted at about middle of head. Antennal scape and pedicel yellow, postpedicel and arista brown (Fig 4G–4I). Antennal pedicel with a few long bristles, pale yellow in *G*. *flavipes* and *G*. *inermis*, dark brown in *G*. *haemorrhoidalis*. Postpedicel small, with the exposed part globular. Lunule pale yellow, bare. Facial plate bare. Parafacial plate yellow, with pale yellow hair-like setae. Gena and genal groove pale yellow, both covered with dense, pale yellow hair-like setae.

#### Thorax

Ground colour dark brown or black (Fig 5), with a yellow, rectangular area near scutoscutellar suture only in *G*. *flavipes* (Figs 3D, 4D and 5A). Postpronotal lobe yellow, distinctly swollen; or sometimes with several tiny dark brown spots in *G*. *inermis*. Hair-like setae mainly yellow, or with a narrow dark brown band on postsutural scutum. In *G*. *haemorrhoidalis*, a mixture of yellow and dark brown or black setae on postalar callus, and the setae anteriorly yellow and posteriorly dark brown on scutellum. Meron brown and bare.

#### Wing

Entirely hyaline in *G*. *flavipes* and *G*. *haemorrhoidalis* (Figs 3A and 3B; 6A and 6B), partly infuscated, with darkened patches with ill-defined edges in *G*. *inermis* (Fig 6C). Tegula and basicosta dark brown. Crossvein r-m situated much closer to the base of the wing than to crossvein dm-cu; distance between crossvein r-m and d-m at least twice as long as r-m; while in *G*. *inermis*, crossvein r-m situated almost at the opposite of crossvein dm-cu.

#### Legs

Totally yellow in *G*. *flavipes*, dark yellow or brown in *G*. *haemorrhoidalis* and *G*. *inermis*, with femora distinctly darkened. Trochanters unarmed, with long, pale yellow hair-like setae. Femora with long, hair-like setae, totally yellow in *G*. *flavipes*, brown anterodorsally and yellow posteroventrally in *G*. *haemorrhoidalis* and *G*. *inermis*. Tibiae setae short, yellow in *G*. *flavipes*, a mixture of yellow and brown setaein *G*. *haemorrhoidalis* and *G*. *inermis*. Hind tibia not swollen (Fig 6D–6F). Hind tarsus with long and strong yellow setae in *G*. *flavipes* and *G*. *haemorrhoidalis*, sparse dorsolaterally and dense ventrolaterally; the setae shorter and sparser in *G*. *inermis*. Tarsal claws yellow at base and dark brown or black at tip, shorter than fifth tarsomere in *G*. *flavipes* and *G*. *inermis*, but approximately as long as the fifth tarsomere in *G*. *haemorrhoidalis*. Pulvilli yellowish.

#### Abdomen

Ground colour mainly yellowish-brown, with a set of variously shaped, sometimes faint and ambiguous dark brown spots dorsally and laterally in *G*. *flavipes* and *G*. *inermis* (Fig 5D and 5F); while in *G*. *haemorrhoidalis*, the colour dark brown or black (Fig 5E). Hair-like setae mainly yellow in *G*. *flavipes* and *G*. *inermis*, yellow anteriorly black in the middle, and reddish-yellow or orangish posteriorly in *G*. *haemorrhoidalis*. Sternites yellow. Male cercus short and broad, length-width ratio less than 1.0; surstylus yellow, gradually tapered proximally and distally, with gradually tapered apex in *G*. *flavipes* (Fig 7A–7C), an abruptly swollen lobe near base dorsally rounded apex in *G*. *haemorrhoidalis* (Fig 7D–7F), and rounded apex in *G*. *inermis* (Fig 7G–7J); surstylar setae long, reaching the sagittal plane in *G*. *flavipes* (Fig 7C), while short, reaching at most halfway to the sagittal plane in *G*. *haemorrhoidalis* (Fig 7F) and *G*. *inermis*.

#### Female

Like the male (Fig 8), except for the following. Width of frons 1/3 width of head in dorsal view (Fig 8G–8I). In *G*. *inermis*, hair-like setae on fronto-orbital and parafacial plate shorter, sparser and darker than the male, forming a dark longitudinal spot at the lower part of parafacial plate on each side (Fig 8I); femur with dark brown or black hair-like setae on the anterior surface of all legs. Abdomen conical, terminalia long and curved forward; segment 7 broader than long; sternite 8 longitudinally ridged in the middle and with scallop-shaped apex (Figs 8D–8F; 9A–9C; 10A–10C; 11A–11C).

#### Egg

Brownish black, posterior part (a continuation of the broad chorionic flanges) elongated as a short and thick pedicel in *G*. *flavipes*, with length-width ratio around 1/4 in lateral view, accounting for 1/3 of the total egg length (Fig 9D); while in *G*. *haemorrhoidalis* the pedicel is long and slender, with length-width ratio around 1/6 in lateral view, accounting for 2/5 of the total egg length (Fig 10D). The egg of *G*. *inermis* yellowish, stalkless, elongate ovoid in shape, with the broad chorionic flanges accounting for 7/10 of the total egg length (Fig 11D).

#### Hosts

Donkey (*Equus africanus asinus* Linnaeus) was given as the host for *G*. *flavipes* by Brauer (1863) without evidence; Burchell’s zebra (*Equus quagga burchellii* Gray), horse [*E*. *ferus caballus* Linnaeus, *E*. *ferus przewalskii* (Poliakov)], and Mongolian wild ass (*E*. *hemionus hemionus* Pallas) are hosts for both *G*. *haemorrhoidalis* and *G*. *inermis*, with donkey and Mountain zebra [*E*. *zebra* (Linnaeus)] as hosts for *G*. *haemorrhoidalis* as well.

#### Distribution

Non-vouchered literature records given with a question mark. So far, *G*. *flavipes* is mainly recorded from the Palaearctic region: China (Inner Mongolia, Xinjiang), Croatia?, Cyprus, Egypt?, France, Iran?, Kazakhstan?, Libya, Morocco, Russia (Siberia), Spain?, Turkey?. *Gasterophilus inermis* is distributed in the Afrotropical, Nearctic and Palaearctic regions, and *G*. *haemorrhoidalis* in all biogeographical regions.

Diagnosis, examined material and distribution of *G*. *flavipes*, *G*. *haemorrhoidalis* and *G*. *inermis* are provided in S1 File.

## Discussion

The distinctive morphology of egg, male terminalia and female ovipositor documented here is considered unequivocal evidence for a specific separation of *G*. *flavipes* and *G*. *haemorrhoidalis*. The original description by Olivier [22] provides only a brief description of the adult, and the name-bearing type from the French Pyrenees could not be located and must be considered as lost. However, the species was widely recognized among the early authors (for references see Brauer [2] and Paramonov [47]), and when Patton [23] provided detailed illustrations of the male terminalia, his aim was not to test the validity of a questionable nominal species but to take advantage of what had become a routine approach of documenting male terminalia to provide users with a more reliable identification tool. Patton [23] noted that his illustrations would “speak for themselves” (p. 351), and that only brief mention would be needed for the diagnostic features of each species. While Patton explicitly pointed to several of the differences also mentioned here, he did not stress the marked difference in surstylar setosity so evident from his illustrations (Patton [23]: Figs 1A and 3A). Patton’s illustrations do not do justice to the equally marked difference in surstylar shape (Fig 7D–7I), which may explain why Zumpt and Paterson [24] and Grunin [3,29] did not recognize *G*. *flavipes* as a valid species.

The COI gene is unable to provide a reliable separation of *G*. *flavipes* from *G*. *haemorrhoidalis* as well as from *G*. *inermis*, which may be seen as evidence of recent divergence [46,48] as has been reported for many other species in Diptera [46,49,50]. It is noteworthy that the genetic distance of the traditional DNA barcode region is higher between *G*. *haemorrhoidalis* and *G*. *inermis* than between *G*. *haemorrhoidalis* and *G*. *flavipes*, which matches the higher degree of morphological similarity between the latter two species. The distribution of *G*. *flavipes* with vouchered records from around the Mediterranean and from Central Asia appears to be more restricted than, but fully sympatric with that of *G*. *haemorrhoidalis*. The type locality for *G*. *flavipes* (French Pyrenees) therefore appears to be in the periphery of its range, but it is here considered not to pose any conflict. A neotype has not been designated, partly as we do not see an exceptional need (as required by ICZN [51]: Article 75.3), and partly because suitable material from or close to the type locality has not been available.

High diversity of equid hosts appears to facilitate the diversification of *Gasterophilus* species [15]. Species diversity and population size of *Gasterophilus* have declined due to the decreased number of other equid reservoirs (e.g. donkeys) and the highly effective and widespread use of anti-parasitic drugs for domestic animals [15,19]. Nevertheless, all the seven Palaearctic *Gasterophilus* species have been recorded in Xinjiang [17,20,21], which represents the highest species diversity of this genus worldwide [3,4,6,15,52,53]. Even more, at least six of these seven species (data unavailable for *G*. *flavipes*) appear to hold stable populations here according to current epidemiological investigations [16,17,21], which makes Xinjiang the world hotspot for *Gasterophilus*. Xinjiang possesses a large area of Gobi Desert where thousands of free-ranging Mongolian asses (*E*. *hemionus hemionus*), semi-captive horses (*E*. *ferus przewalskii*), and domestic horses (*E*. *ferus caballus*) and donkeys (*E*. *africanus asinus*) are distributed [16,17,21], which provide more sustainable environment and a larger host carrying capacity than in the western Palaearctic, where populations of *Gasterophilus* appear to be in decline [15]. Considering that species richness of obligate parasites is tightly correlated with that of their hosts [54,55], we suggest that the availability of a diverse equid fauna in Xinjiang is the main reason for the high number of *Gasterophilus* species. The high number of free-grazing equids (e.g. Mongolian asses and Przewalski's horse), which are free from anti-parasitic treatments, may be an important factor in maintaining the high number of *Gasterophilus* species in Xinjiang as well [15,19].

Our study shows that morphological characters are still indispensable for a reliable species-level identification of adult *Gasterophilus* specimens. The primary field investigation suggests that *G*. *flavipes*, which takes up 62% of the *Gasterophilus* samples collected by Malaise trap, is prevalent in Xinjiang. The confusion between the overlooked *G*. *flavipes* and the morphologically and molecularly similar *G*. *haemorrhoidalis* and *G*. *inermis* calls for more careful veterinarian investigations and a re-assessment of the epidemiology of gasterophilosis in areas where *G*. *flavipes* is known or supposed to occur.

## Supporting information

S1 Table Specimen information, molecular markers, GenBank accession numbers, collecting localities and references for *Gasterophilus* species. Collecting localities are given with country and province.
(DOCX)

S2 Table Intraspecific genetic divergences (using K2P model) and standard error estimate(s) (1000 bootstrap replicates) of the traditional barcode region (670-bp region near the 5' terminus of COI) in *Gasterophilus* species. (DOCX)

S3 Table Intraspecific genetic divergences (using K2P model) and standard error estimate(s) (1000 replicates of bootstrap) of a 663-bp region near the 3' terminus of COI in the seven Palaearctic species of *Gasterophilus*. (DOCX)

S4 Table Interspecific percentage genetic divergences (using K2P model) of the traditional DNA barcode region (670-bp region near the 5' terminus of COI) in *Gasterophilus* species. Standard error estimate(s) (1000 bootstrap replicates) are shown under the diagonal. (DOCX)

S5 Table Interspecific percentage genetic divergences (using K2P model) of a 663-bp region near the 3' terminus of COI in *Gasterophilus* species. Standard error estimate(s) (1000 bootstrap replicates) are shown under the diagonal.
(DOCX)

S1 File Diagnosis, examined material and distribution of *Gasterophilus flavipes*, *G*. *haemorrhoidalis* and *G*. *inermis*. (PDF)

## References

[1] Brauer F. Neue Beiträge zur Kenntniss der europäischen Oestriden. Verhandlungen der Kais Zool Gesellschaft Wien. 1858;8: 464.

[2] Brauer F. Monographie der Oestriden. Wien: W. Braumüller; 1863.

[3] Grunin KJ. Gasterophilidae In: Lindner E., editor. Die Fliegen der Paläarktischen Region. Stuttgart: Schweizerbart’sche; 1969 pp. 1–66.

[4] Zumpt F. Myiasis of Man and Animals in the Old World. London: Butterworths Ltd; 1965.

[5] Papavero N. The world Oestridae (Diptera) mammals and continental drift. Ser Entomol. 1977;14: 1–240.

[6] Colwell DD, Hall MJR, Scholl PJ. The oestrid flies: biology, host-parasite relationships, impact and management. Colwell DD, Hall MJR, Scholl PJ, editors. Wallingford: CABI; 2006 10.1079/9780851996844.0000

[7] Pont AC. Familiy Oestridae In: Delfinado M, Hardy D, editors. A Catalog of the Diptera of the Oriental Region: Suborder Cyclorrhapha. Honolulu: University Press of Hawaii; 1973 pp. 700–701.

[8] Soós À, Minář J. Family Gasterophilidae In: Soós À, Papp L, editors. Catalogue of Palaearctic Diptera, Vol 11 Scathophagidae—Hypodermatidae. Amsterdam: Elsevier; 1986 pp. 237–240.

[9] Wood DM. Oestridae In: McAlpine JF, editor. Manual of Nearctic Diptera, Vol 2. Ottawa: Research Branch, Agriculture Canada; 1987 pp. 1147–1158.

[10] Hall M, Wall R. Myiasis of humans and domestic animals. Adv Parasitol. 1995;35: 257–334. 10.1016/S0065-308X(08)60073-1 7709854

[11] Sequeira JL, Tostes RA, Oliveira-Sequeira TCG. Prevalence and macro- and microscopic lesions produced by *Gasterophilus nasalis* (Diptera: Oestridae) in the Botucatu Region, SP, Brazil. Vet Parasitol. 2001;102: 261–266. 10.1016/S0304-4017(01)00536-2 11777606

[12] Wood DM. Morphology of Adult Oestridae In: Colwell DD, Hall MJR, Scholl PJ, editors. The oestrid flies: biology, host-parasite relationships, impact and management. Wallingford: CABI; 2006 pp. 79–80.

[13] Bezdekova B, Jahn P, Vyskocil M. Pathomorphological study on gastroduodenal ulceration in horses: Localisation of lesions. Acta Vet Hung. 2007;55: 241–249. 10.1556/AVet.55.2007.2.10 17555289

[14] Getachew AM, Innocent G, Trawford AF, Reid SWJ, Love S. Gasterophilosis: A major cause of rectal prolapse in working donkeys in Ethiopia. Trop Anim Health Prod. 2012;44: 757–762. 10.1007/s11250-011-9961-7 21870062

[15] Otranto D, Milillo P, Capelli G, Colwell DD. Species composition of *Gasterophilus* spp. (Diptera, Oestridae) causing equine gastric myiasis in southern Italy: Parasite biodiversity and risks for extinction. Vet Parasitol. 2005;133: 111–118. 10.1016/j.vetpar.2005.05.015 15978726

[16] Huang H, Zhang B, Chu H, Zhang D, Li K. *Gasterophilus* (Diptera, Gasterophilidae) infestation of equids in the Kalamaili Nature Reserve, China. Parasite. 2016. 10.1051/parasite/2016036 PMC5018932 27593434

[17] Liu SH, Li K, Hu DF. The incidence and species composition of *Gasterophilus* (Diptera, Gasterophilidae) causing equine myiasis in northern Xinjiang, China. Vet Parasitol. 2016;217: 36–38. 10.1016/j.vetpar.2015.12.028 26827858

[18] Pawlas-Opiela M, Wojciech Ł, Sołtysiak Z, Otranto D, Ugorski M. Molecular comparison of *Gasterophilus intestinalis* and *Gasterophilus nasalis* from two distinct areas of Poland and Italy based on cox1 sequence analysis. Vet Parasitol. 2010;169: 219–221. 10.1016/j.vetpar.2009.12.030 20080351

[19] Colwell DD, Otranto D, Stevens JR. Oestrid flies: eradication and extinction versus biodiversity. Trends Parasitol. 2009;25: 500–504. 10.1016/j.pt.2009.07.011 19762281

[20] Huang H, Chu H, Cao J, Bu L, Hu D, Zhang D, et al Distribution of *Gasterophilus* (Diptera, Gasterophilidae) Myiasis Foci in Arid Desert Steppe: a case study of Kalamaili Mountain Ungulate Nature Reserve. Linye Kexue/Scientia Silvae Sin. 2017;53 10.11707/j.1001-7488.20171116

[21] Li X, Chen Y, Wange Q, Li K, Pape T, Dong Z. Molecular and morphological characterization of third instar Palaearctic horse stomach bot fly larvae (Oestridae: Gasterophilinae, *Gasterophilus*). Vet Parasitol. 2018;262: 56–74. 10.1016/j.vetpar.2018.09.011 30389013

[22] Olivier GA. Encyclopédie méthodique. Histoire naturelle. Insectes. Paris: Histoire naturelle; 1811.

[23] Patton WS. Studies on the higher Diptera of medical and veterinary importance. Ann Trop Med Parasitol. 1937;31: 351–359.

[24] Zumpt F, Paterson HE. Studies on the family Gasterophilidae, with keys to the adults and maggots. J Entomol Soc South Afr. 1953;16: 59–72.

[25] Pape T. Phylogeny of Oestridae (Insecta: Diptera). Syst Entomol. 2001;26: 133–171. 10.1046/j.1365-3113.2001.00143.x

[26] Cumming JM, Wood DM. Adult morphology and terminology In: Brown BV, Borkent A, Cumming JM, editors. Manual of Central American Diptera Vol1. Ottawa: NRC Research Press; 2009 pp. 9–50.

[27] Guimarães JH. Family Gasterophilidae. A Catalogue of the Diptera of the Americas South of the United States. São Paulo: Departamento de Zoologia, Secretaria da Agricultura; 1967 pp. 1–2.

[28] Guimarães JH, Papavero N. Myiasis caused by obligatory parasites. III. *Gasterophilus* Leach (Gasterophilidae). Myiasis in Man and Animals in the Neotropical Region; Bibliographic Database. São Paulo: Editora Plêiade; 1999 pp. 167–173.

[29] Grunin KJ. Gastrophilidae In: Lindner E., editor. Fauna URSS, Insecta: Diptera. Moscow: Education of Academy of Sciences of USSR; 1955 pp. 1–96.

[30] James MT. The flies that cause myiasis in man. Washington: United States Goverment Printing Office; 1974.

[31] Pont AC. Family Gasterophilidae In: Crosskey RW, editor. Catalogue of the Diptera of the Afrotropical Region. British Museum of Natural History; 1980 pp. 883–884.

[32] Kaboret Y, Pangui LJ, Vercruysse J. Note on gasterophilosis in donkeys in Burkina Faso. Rev Elev Med Vet Pays Trop. 1986;39. 3589069

[33] Pearse B, Peucker S, Cottam R. Identification of *Gasterophilus haemorrhoidalis* in Queensland. Aust Vet J. 1989;66: 380 10.1111/j.1751-0813.1989.tb09745.x 2619658

[34] Xue WQ, Wang MF. Gasterophilidae. Flies of ChinaVol2. Shenyang: Liaoning Science and Technology Press; 1996 pp. 2207–2215.

[35] Tavassoli M, Bakht M. *Gastrophilus* spp. myiasis in Iranian equine. Sci Parasitol. 2012;13: 83–86.

[36] Mashayekhi M, Ashtari B. Study of *Gasterophilus* role in Equine Gastric Ulcer Syndrome in Tabriz area. Bull Environ Pharmacol Life Sci. 2013;2: 169–172.

[37] de Jong Y, Verbeek M, Michelsen V, Bjørn P de P, Los W, Steeman F, et al Fauna Europaea–all European animal species on the web. Biodivers Data J. 2014;2: e4034 10.3897/BDJ.2.e4034 25349527 PMC4206781

[38] Folmer O, Black M, Hoeh W, Lutz R, Vrijenhoek R. DNA primers for amplification of mitochondrial cytochrome c oxidase subunit I from diverse metazoan invertebrates. Hansson B, editor. Mol Mar Biol Biotechnol. 1994;3: 294–299. 10.1371/journal.pone.0013102 7881515

[39] Nelson LA, Wallman JF, Dowton M. Using COI barcodes to identify forensically and medically important blowflies. Med Vet Entomol. 2007;21: 44–52. 10.1111/j.1365-2915.2007.00664.x 17373946

[40] Jordaens K, Sonet G, Richet R, Dupont E, Braet Y, Desmyter S. Identification of forensically important *Sarcophaga* species (Diptera: Sarcophagidae) using the mitochondrial COI gene. Int J Legal Med. 2013;127: 491–504. 10.1007/s00414-012-0767-6 22960880

[41] Zhang D, Yan L, Zhang M, Chu H, Cao J, Li K, et al Phylogenetic inference of calyptrates, with the first mitogenomes for Gasterophilinae (Diptera: Oestridae) and Paramacronychiinae (Diptera: Sarcophagidae). Int J Biol Sci. 2016;12: 489–504. 10.7150/ijbs.12148 27019632 PMC4807417

[42] Kutty SN, Pape T, Wiegmann BM, Meier R. Molecular phylogeny of the Calyptratae (Diptera: Cyclorrhapha) with an emphasis on the superfamily Oestroidea and the position of Mystacinobiidae and McAlpine’s fly. Syst Entomol. 2010;35: 614–635. 10.1111/j.1365-3113.2010.00536.x

[43] Katoh K, Standley DM. MAFFT multiple sequence alignment software version 7: Improvements in performance and usability. Mol Biol Evol. 2013;30: 772–780. 10.1093/molbev/mst010 23329690 PMC3603318

[44] Kumar S, Stecher G, Li M, Knyaz C, Tamura K. MEGA X: Molecular evolutionary genetics analysis across computing platforms. Mol Biol Evol. 2018;35: 1547–1549. 10.1093/molbev/msy096 29722887 PMC5967553

[45] Nei M, Kumar S. Molecular Evolution and Phylogenetics New York: Oxford University Press; 2000.

[46] Meier R, Shiyang K, Vaidya G, Ng PKL. DNA barcoding and taxonomy in diptera: A tale of high intraspecific variability and low identification success. Syst Biol. 2006;55: 715–728. 10.1080/10635150600969864 17060194

[47] Paramonov SJ. Flies of the genus *Gastrophilus* and their control. Kiev: Kiev: Acad. des Sci. Zoo; 1940.

[48] Meier R, Zhang G, Ali F. The use of mean instead of smallest interspecific distances exaggerates the size of the “barcoding gap” and leads to misidentification. Syst Biol. 2008;57: 809–813. 10.1080/10635150802406343 18853366

[49] Buenaventura E, Valverde-Castro C, Wolff M, Triana-Chavez O, Gómez-Palacio A. DNA barcoding for identifying synanthropic flesh flies (Diptera, Sarcophagidae) of Colombia. Acta Trop. 2018;182: 291–297. 10.1016/j.actatropica.2018.01.020 29408406

[50] Meiklejohn KA, Wallman JF, Dowton M. DNA-based identification of forensically important Australian Sarcophagidae (Diptera). Int J Legal Med. 2011;125: 27–32. 10.1007/s00414-009-0395-y 19997851

[51] ICZN. International Code of Zoological Nomenclature. 4th ed Lindner E, editor. Stuttgart: Schweizerbart’sche; 1999.

[52] Felix SR, Silva CE, Schmidtt E, Nizoli LQ, Götze MM, Silva SS. Presence of *Gasterophilus* (Leach, 1817) (Diptera: Oestridae) in horses in Rio Grande do Sul State, Brazil. Parasitol Latinoam. 2007;62: 122–126.

[53] Pilo C, Altea A, Scala A. Gasterophilosis in horses in Sardinia (Italy): effect of meteorological variables on adult egg-laying activity and presence of larvae in the digestive tract, and update of species. Parasitol Res. 2015;114: 1693–1702. 10.1007/s00436-015-4352-z 25663068

[54] Lafferty KD. Biodiversity loss decreases parasite diversity: Theory and patterns. Philosophical Transactions of the Royal Society B: Biological Sciences. 2012 pp. 2814–2827. 10.1098/rstb.2012.0110 22966137 PMC3427564

[55] Kamiya T, O’Dwyer K, Nakagawa S, Poulin R. Host diversity drives parasite diversity: meta-analytical insights into patterns and causal mechanisms. Ecography (Cop). 2014;37: 689–697.

