# Supplementary material for: Gasterophilus flavipes (Oestridae: Gasterophilinae): A horse stomach bot fly brought back from oblivion with morphological and molecular evidence
Source: PLoS One. 2019 Aug 12;14(8):e0220820. doi: 10.1371/journal.pone.0220820 (PMC6690546; doi:10.1371/journal.pone.0220820)
Supplement: S1 Table — Collecting localities are given with country and province. (DOCX) [file pone.0220820.s001.docx]

**S1 Table. Specimen information, molecular markers, GenBank accession numbers, collecting localities and references for *Gasterophilus* species. Collecting localities are given with country and province.**

| **Name** | **Molecular**  **marker** | **Genbank** | **Country** | **Province Reference** | |
| --- | --- | --- | --- | --- | --- |
| *G. flavipes* 1 | cox1 | MK412087.1 | China | Xinjiang | Present study |
| *G. flavipes* 2 | cox1 | MK412088.1 | China | Xinjiang | Present study |
| *G. flavipes* 3 | cox1 | MK412089.1 | China | Xinjiang | Present study |
| *G. haemorrhoidalis* MH553415  *G. haemorrhoidalis* MH553434 | 5’ cox1  3’ cox1 | MH553415.1  MH553434.1 | China | Xinjiang | Li et al. (2018) |
| *G. haemorrhoidalis* MH553416  *G. haemorrhoidalis* MH553435 | 5’ cox1  3’ cox1 | MH553416.1  MH553435.1 | China | Xinjiang | Li et al. (2018) |
| *G. haemorrhoidalis* MH553417  *G. haemorrhoidalis* MH553436 | 5’ cox1  3’ cox1 | MH553417.1  MH553436.1 | China | Xinjiang | Li et al. (2018) |
| *G. haemorrhoidalis* MH553418  *G. haemorrhoidalis* MH553437 | 5’ cox1  3’ cox1 | MH553418.1  MH553437.1 | Mongolia | – | Li et al. (2018) |
| *G. haemorrhoidalis* AF497774 | 3’ cox1 | AF497774.1.1 | Italy | Apulia | Otranto et al. (2003) |
| *G. haemorrhoidalis* Gh | 3’ cox1 | – | Italy | Apulia | Otranto et al. (2005) |
| *G. inermis* MH553413  *G. inermis* MH553432 | 5’ cox1  3’ cox1 | MH553413.1  MH553432.1 | China | Xinjiang | Li et al. (2018) |
| *G. inermis* MH553414  *G. inermis* MH553433 | 5’ cox1  3’ cox1 | MH553414.1  MH553433.1 | Mongolia | – | Li et al. (2018) |
| *G. inermis* Gine | 3’ cox1 | – | Italy | Apulia | Otranto et al. (2005) |
| *G. intestinalis* MH553410  *G. intestinalis* MH553411 | 5’ cox1  3’ cox1 | MH553410.1  MH553429.1 | China | Xinjiang | Li et al. (2018) |
| *G. intestinalis* MH553411  *G. intestinalis* MH553430 | 5’ cox1  3’ cox1 | MH553411.1  MH553430.1 | China | Xinjiang | Li et al. (2018) |
| *G. intestinalis* MH553412  *G. intestinalis* MH553431 | 5’ cox1  3’ cox1 | MH553412.1  MH553431.1 | China | Qinghai | Li et al. (2018) |
| *G. intestinalis* KU236026 | 3’ cox1 | KU236026.1 | China | Heilongjiang | Gao et al. (2016) |
| *G. intestinalis* KU236025 | 3’ cox1 | KU236025.1 | China | Xinjiang | Gao et al. (2016) |
| *G. intestinalis* GU265748 | 3’ cox1 | GU265748.1 | Poland | Rawicz | Pawlas–Opiela et al. (2010) |
| *G. intestinalis* GU299283 | 3’ cox1 | GU299283.1 | Italy | Apulia | Pawlas–Opiela et al. (2010) |
| *G. intestinalis* KX548232 | 3’ cox1 | KX548232.1 | Italy | – | Cavallero et al. (2017) |
| *G. intestinalis* AF257117 | 3’ cox1 | AF257117.1 | Italy | Apulia | Otranto et al. (2003) |
| *G. intestinalis* Gi | 3’ cox1 | – | Italy | Apulia | Otranto et al. (2005) |
| *G. nasalis* MH553408  *G. nasalis* MH553426 | 5’ cox1  3’ cox1 | MH553408.1  MH553426.1 | China  China | Xinjiang  Xinjiang | Li et al. (2018)  Li et al. (2018) |
| *G. nasalis* MH553409  *G. nasalis* MH553427 | 5’ cox1  3’ cox1 | MH553409.1  MH553427.1 | China | Xinjiang | Li et al. (2018) |
| *G. nasalis* AF497775 | 3’ cox1 | AF497775.1 | Italy | Apulia | Otranto et al. (2003) |
| *G. nasalis* KM087716 | 3’ cox1 | KM087716.1 | China | Xinjiang | Zhang et al. (2018) |
| *G. nasalis* GU265757 | 3’ cox1 | GU265757.1 | Poland | Rawicz | Pawlas–Opiela et al. (2010) |
| *G. nasalis* GU299286 | 3’ cox1 | GU299286.1 | Italy | Apulia | Pawlas–Opiela et al. (2010) |
| *G. nasalis* Gn | 3’ cox1 | – | Italy | Apulia | Otranto et al. (2005) |
| *G. nigricornis* MH553407  *G. nigricornis* MH553421 | 5’ cox1  3’ cox1 | MH553407.1  MH553421.1 | China | Xinjiang | Li et al. (2018) |
| *G. nigricornis* MH553404  *G. nigricornis* MH553423 | 5’ cox1  3’ cox1 | MH553404.1  MH553423.1 | China | Xinjiang | Li et al. (2018) |
| *G. nigricornis* MH553406  *G. nigricornis* MH553425 | 5’ cox1  3’ cox1 | MH553406.1  MH553425.1 | China | Qinghai | Li et al. (2018) |
| *G. nigricornis* MH553405  *G. nigricornis* MH553424 | 5’ cox1  3’ cox1 | MH553405.1  MH553424.1 | Mongolia | – | Li et al. (2018) |
| *G. nigricornis* MH553403  *G. nigricornis* MH553422 | 5’ cox1  3’ cox1 | MH553403.1  MH553422.1 | Mongolia | – | Li et al. (2018) |
| *G. nigricornis* KM087740 | 3’ cox1 | KM087740.1 | China | Xinjiang | Zhang et al. (2018) |
| *G. pecorum* MH553401  *G. pecorum* MH553419 | 5’ cox1  3’ cox1 | MH553401.1  MH553419.1 | China | Xinjiang | Li et al. (2018) |
| *G. pecorum* MH553402  *G. pecorum* MH553420 | 5’ cox1  3’ cox1 | MH553402.1  MH553420.1 | China | Xinjiang | Li et al. (2018) |
| *G. pecorum* KF844358 | 3’ cox1 | KF844358.1 | China | Xinjiang | Wang et al. (2014) |
| *G. pecorum* KU578262 | 3’ cox1 | KU578262.1 | China | Xinjiang | Zhang et al. (2016) |
| *G. pecorum* AF497776 | 3’ cox1 | AF497776.1 | Italy | Apulia | Otranto et al. (2003) |
| *G. pecorum* Gp | 3’ cox1 | – | Italy | Apulia | Otranto et al. (2005) |
| *Hypoderma lineatum* GU584123 | cox1 | GU584123.1 | Italy | Basilicata | Weigl et al. (2010) |
| *Dermatobia hominis* AY463155 | cox1 | AY463155.1 | – | – | Unpublished |

References (cited in the Tables but not referred in the main text):

Cavallero, S., Pombi, M., Perrone, V., Milardi, G. L., D’Amelio, S., Giuliani, C., Gabrielli, S., 2017. *Gasterophilus* *intestinalis* (Diptera: Oestridae) in the diaphragmatic muscle: An unusual finding. Vet. Parasitol. 237, 117–121.

Gao, D.Z., Liu, G.H., Song, H.Q., Wang, G.L., Wang, C.R., Zhu, X.Q., 2016. The complete mitochondrial genome of *Gasterophilus* *intestinalis*, the first representative of the family Gasterophilidae. Parasitol. Res. 115, 2573–2579.

Gao, D.Z., Liu, G.H., Zhu, X.Q., Wang, C.R., 2015. Genetic variation of *Gasterophilus* *intestinalis* (Insecta: Oestridae) in three mitochondrial DNA sequences originating from Heilongjiang Provience and Xinjiang Uygur Autonomous Region, China. The 13^th^ Meeting of Chinese Association of Animal Science and Veterinary Medicine, Lanzhou, Gansu Province, China 276.

Nirmala, X., Hypša, V., Žurovec, M., 2001. Molecular phylogeny of Calyptratae (Diptera: Brachycera): the evolution of 18S and 16S ribosomal rDNAs in higher dipterans and their use in phylogenetic inference. Insect. Mol. Biol. 10, 475–485.

Otranto, D., Traversa, D., Guida, B., Tarsitano, E., Fiorente, P., & Stevens, J.R., 2003. Molecular characterization of the mitochondrial cytochrome oxidase I gene of Oestridae species causing obligate myiasis. Med. Vet. Entomol. 17, 307–315.

Pawlas-Opiela, M., Wojciech, Ł., Sołtysiak, Z., Otranto, D., Ugorski, M., 2010. Molecular comparison of *Gasterophilus* *intestinalis* and *Gasterophilus* *nasalis* from two distinct areas of Poland and Italy based on cox1 sequence analysis. Vet. Parasitol. 169, 219–221.

Wang, W., Zhang, D., Hu, D., Chu, H., Cao, J., Ente, M., Jiang, G., Li, K., 2014. Population genetic structure of *Gasterophilus* *pecorum* in the Kalamaili Nature Reserve, Xinjiang, based on mitochondrial cytochrome oxidase (COI) gene sequence. Med. Vet. Entomol. 28, 75–82.

Weigl, S., Testini, G., Parisi, A., Dantas-Torres, F., Traversa, D., Colwell, D.D., Otranto, D., 2010. The mitochondrial genome of the common cattle grub, *Hypoderma* *lineatum*. Med. Vet. Entomol. 24, 329–335.

Zhang, B., Huang, H., Wang, H., Zhang, D., Chu, H., Ma, X., Ge, Y., Ente, M., Li, K., 2018. Genetic diversity of common *Gasterophilus* spp. from distinct habitats in China. Parasit. Vectors 11, 474.
