# Supplementary material for: Gasterophilus flavipes (Oestridae: Gasterophilinae): A horse stomach bot fly brought back from oblivion with morphological and molecular evidence
Source: PLoS One. 2019 Aug 12;14(8):e0220820. doi: 10.1371/journal.pone.0220820 (PMC6690546; doi:10.1371/journal.pone.0220820)
Supplement: S2 Table — (DOCX) [file pone.0220820.s002.docx]

**S2 Table. Intraspecific genetic divergences (using K2P model) and standard error estimate(s) (1000 bootstrap replicates) of the traditional barcode region (670-bp region near the 5' terminus of COI) in *Gasterophilus* species.**

| Species | Distance | S.E. |
| --- | --- | --- |
| *Gasterophilus flavipes* | 0.0010 | 0.0010 |
| *Gasterophilus haemorrhoidalis* | 0.0110 | 0.0026 |
| *Gasterophilus inermis* | 0.0142 | 0.0045 |
| *Gasterophilus intestinalis* | 0.0204 | 0.0035 |
| *Gasterophilus nasalis* | 0.0095 | 0.0037 |
| *Gasterophilus nigricornis* | 0.0129 | 0.0033 |
| *Gasterophilus pecorum* | 0.0063 | 0.0026 |
