# Supplementary material for: Gasterophilus flavipes (Oestridae: Gasterophilinae): A horse stomach bot fly brought back from oblivion with morphological and molecular evidence
Source: PLoS One. 2019 Aug 12;14(8):e0220820. doi: 10.1371/journal.pone.0220820 (PMC6690546; doi:10.1371/journal.pone.0220820)
Supplement: S3 Table — (DOCX) [file pone.0220820.s003.docx]

**S3 Table. Intraspecific genetic divergences (using K2P model) and standard error estimate(s) (1000 replicates of bootstrap) of a 663-bp region near the 3' terminus of COI in the seven Palaearctic species of *Gasterophilus*.**

| Species | Distance | S.E. |
| --- | --- | --- |
| *Gasterophilus flavipes* | 0.0010 | 0.0010 |
| *Gasterophilus haemorrhoidalis* | 0.0030 | 0.0015 |
| *Gasterophilus inermis* | 0.0076 | 0.0032 |
| *Gasterophilus intestinalis* | 0.0119 | 0.0022 |
| *Gasterophilus nasalis* | 0.0179 | 0.0031 |
| *Gasterophilus nigricornis* | 0.0179 | 0.0038 |
| *Gasterophilus pecorum* | 0.0036 | 0.0051 |
