# Supplementary material for: Gasterophilus flavipes (Oestridae: Gasterophilinae): A horse stomach bot fly brought back from oblivion with morphological and molecular evidence
Source: PLoS One. 2019 Aug 12;14(8):e0220820. doi: 10.1371/journal.pone.0220820 (PMC6690546; doi:10.1371/journal.pone.0220820)
Supplement: S4 Table — (DOCX) [file pone.0220820.s004.docx]

**S4 Table. Interspecific percentage genetic divergences (using K2P model) of the traditional DNA barcode region (670-bp region near the 5' terminus of COI) in *Gasterophilus* species. Standard error estimate(s) (1000 bootstrap replicates) are shown under the diagonal.**

| No. | Species | 1 | 2 | 3 | 4 | 5 | 6 | 7 |
| --- | --- | --- | --- | --- | --- | --- | --- | --- |
| 1 | *Gasterophilus flavipes* |  | 0.0067 | 0.0068 | 0.2243 | 0.2121 | 0.2038 | 0.2252 |
| 2 | *Gasterophilus haemorrhoidalis* | 0.0022 |  | 0.0111 | 0.2211 | 0.2128 | 0.2073 | 0.2257 |
| 3 | *Gasterophilus inermis* | 0.0022 | 0.0025 |  | 0.2229 | 0.2145 | 0.2072 | 0.2276 |
| 4 | *Gasterophilus intestinalis* | 0.0208 | 0.0203 | 0.0205 |  | 0.1863 | 0.1876 | 0.1835 |
| 5 | *Gasterophilus nasalis* | 0.0195 | 0.0192 | 0.0194 | 0.0172 |  | 0.1554 | 0.1859 |
| 6 | *Gasterophilus nigricornis* | 0.0195 | 0.0194 | 0.0196 | 0.0170 | 0.0155 |  | 0.1912 |
| 7 | *Gasterophilus pecorum* | 0.0201 | 0.0197 | 0.0199 | 0.0169 | 0.0168 | 0.0186 |  |
