# Supplementary material for: Gasterophilus flavipes (Oestridae: Gasterophilinae): A horse stomach bot fly brought back from oblivion with morphological and molecular evidence
Source: PLoS One. 2019 Aug 12;14(8):e0220820. doi: 10.1371/journal.pone.0220820 (PMC6690546; doi:10.1371/journal.pone.0220820)
Supplement: S5 Table — Standard error estimate(s) (1000 bootstrap replicates) are shown under the diagonal. (DOCX) [file pone.0220820.s005.docx]

**S5 Table. Interspecific percentage genetic divergences (using K2P model) of a 663-bp region near the 3' terminus of COI in *Gasterophilus* species. Standard error estimate(s) (1000 bootstrap replicates) are shown under the diagonal.**

| No. | Species | 1 | 2 | 3 | 4 | 5 | 6 | 7 |
| --- | --- | --- | --- | --- | --- | --- | --- | --- |
| 1 | *Gasterophilus flavipes* |  | 0.0028 | 0.0044 | 0.1558 | 0.1652 | 0.1862 | 0.2073 |
| 2 | *Gasterophilus haemorrhoidalis* | 0.0014 |  | 0.0045 | 0.1545 | 0.1653 | 0.1839 | 0.2059 |
| 3 | *Gasterophilus inermis* | 0.0017 | 0.0017 |  | 0.1521 | 0.1669 | 0.1825 | 0.2057 |
| 4 | *Gasterophilus intestinalis* | 0.0160 | 0.0159 | 0.0157 |  | 0.1699 | 0.1609 | 0.1787 |
| 5 | *Gasterophilus nasalis* | 0.0162 | 0.0161 | 0.0160 | 0.0170 |  | 0.1506 | 0.2029 |
| 6 | *Gasterophilus nigricornis* | 0.0181 | 0.0178 | 0.0176 | 0.0161 | 0.0154 |  | 0.1713 |
| 7 | *Gasterophilus pecorum* | 0.0183 | 0.0181 | 0.0180 | 0.0169 | 0.0182 | 0.0168 |  |
