# Supplementary material for: Gasterophilus flavipes (Oestridae: Gasterophilinae): A horse stomach bot fly brought back from oblivion with morphological and molecular evidence
Source: PLoS One. 2019 Aug 12;14(8):e0220820. doi: 10.1371/journal.pone.0220820 (PMC6690546; doi:10.1371/journal.pone.0220820)
Supplement: S1 File — (PDF) [file pone.0220820.s006.pdf]

S1 File. Diagnosis, examined material and distribution of *Gasterophilus flavipes*, *G. haemorrhoidalis* and *G. inermis*.

Abbreviations for institutions:

IOZ Institute of Zoology, Chinese Academy of Sciences, Beijing, China

MBFU Beijing Forestry University, Beijing, China

MNHN Museum National d'Histoire Naturelle, Paris, France

NHMUK Natural History Museum, London, England

NHMD Natural History Museum of Denmark, University of Copenhagen, Denmark

NHMW Naturhistorisches Museum, Wien, Austria

Gasterophilus flavipes

Diagnosis. Facial plate bare. Postsutural scutum with a light colour (yellowish), rectangular area near scutoscutellar suture. Wing completely hyaline. Crossvein r-m situated much closer to the base of the wing than to crossvein dm-cu; distance between crossvein r-m and dm-cu at least twice as long as r-m. Meron bare. Legs yellow; hind tarsus with long, strong and dense setae ventrolaterally. Abdomen ground colour yellow. Male cercus short and broad, length-width ratio less than 1.0; surstylus yellow, gradually tapered proximally and distally, with gradually tapered apex; surstylar setae long, reaching the sagittal plane. Egg brownish black, posterior part elongated as a short and thick pedicel (a continuation of the broad chorionic flanges), with length-width ratio around 1/4, accounting for 1/3 of the total egg length.

Material examined. 11♂, 15♀, CHINA: Xinjiang Uyghur Autonomous Region, Kalamaili, Qiaomuxibai water reservoir, 1000 m (45°13’48”N 89°03’00”E), 26.VI.2017, Y.-q. Ge & W.-y. Pei (10♂, 14♀ in MBFU; 1♂, 1♀ in NHMD); 1♂, Inner Mongolia, Chifeng (43º20’12”N; 118º39’29”E), 1.VII.1960, collector unknown (IOZ); 1♂, Inner Mongolia, Chifeng, 3.VII.1960, collector unknown (IOZ). MOROCCO: 1♂, 1897, G. Buchet leg. (MNHN); 1♂, Haute Moulouya, 1918, Thullet leg. (MNHN); 1♂, Moyen Atlas, VI.1949, L. Chopard leg. (MNHN). 1♂, CYPRUS: no further data (NHMUK). 1♀, LIBYA: Zuwarah, no further data (NHMUK). 1♂, SUDAN: Dueim, 1937, collector unknown (NHMUK). 1♂, no further data (NHMUK). RUSSIA: 1♂, Siberia, no further data (NHMD).

Hosts. Donkey (*Equus africanus asinus* Linnaeus) [(indicated by Brauer (1863) without evidence].

Distribution. **Afrotropical**–Sudan. **Palaearctic**–China (Inner Mongolia, Xinjiang), Croatia?, Cyprus, Egypt?, France, Iran?, Kazakhstan?, Libya, Morocco, Russia (Siberia), Spain?, Turkey?

Remarks. Olivier [1] did not indicate the number of specimens upon which his description of male *Oestrus flavipes* was

based and no material was recovered in the MNHN, where only a name label is left. Brauer [2] recorded *G. flavipes* from Croatia ("Dalmatia"), Spain, Egypt ("Abukir", i.e., Abu Qir in Alexandria) and Turkey ("Amasia", i.e., Amasya in northern Turkey). Zumpt and Paterson [3] considered *G. flavipes* as a synonym of *G. haemorrhoidalis* mainly based on the illustrations in Patton [4]. Grunin [5] treated *G. flavipes* as a variety ("var.") of *G. haemorrhoidalis*, adding Iran, Kazakhstan and Sudan [5], but he later Grunin [6] treated this nominal taxon as a synonym with no further remarks. The distribution of non-vouchered literature records is provided with question marks.

***Gasterophilus haemorrhoidalis* (Linnaeus)**

Diagnosis. Facial plate bare. Wing completely hyaline. Crossvein dm-cu situated much further away from the base of the wing than crossvein r-m; distance between crossvein r-m and dm-cu at least twice as long as r-m. Meron bare. Legs yellowish brown, with femora distinctly darkened; hind tarsus with long, strong and dense setae ventrolaterally. Abdomen ground colour dark brown or black. Male cercus short and broad, length-width ratio less than 1.0; surstylus yellow, with an abruptly swollen lobe near base and rounded apex; surstylar setae short, reaching at most halfway to the sagittal plane. Egg brownish black, posteriorly part elongated as a long and slender pedicel (a continuation of the broad chorionic flanges), with length-width ratio around 1/6, accounting for 2/5 of the total egg length.

Material examined. CHINA: Inner Mongolia: 20♂, 11♀, Chifeng, Zhaowuda League, Right Banner, 20.V.–17.IX.1960, collector unknown (IOZ); 1♂, Ulanqab, Temurtei, 5.VI.1971, collector unknown (IOZ); 1♀, Xilingol League, Dongwu Banner, 24.VIII.1971, collector unknown (IOZ). Heilongjiang Province: 1♂, Anda, 26.VII.1965, collector unknown (IOZ); 1♀, Qiqihar, Fuyu County, 8.VIII.1966, collector unknown (IOZ). Xinjiang Uyghur Autonomous Region: 1♂, Wusu, 2000 m, 11.VI.1971 (IOZ); 1♀, Xinjiang Uyghur Autonomous Region, Kalamaili, 3.IV.2011, D. Zhang leg. (MBFU); 1♂, Xinjiang Uyghur Autonomous Region, Kalamaili, 6.V.2011, D. Zhang leg. (MBFU).

Hosts. Burchell's zebra (*Equus quagga burchellii* Gray), donkey (*E. africanus asinus* Linnaeus), horse [*E. ferus caballus* Linnaeus, *E. ferus przewalskii* (Poliakov)], Mongolian wild ass (*E. hemionus hemionus* Pallas), Mountain zebra [*E. zebra* (Linnaeus)].

Distribution. **Afrotropical**–Democratic Republic of the Congo, Ethiopia, Kenya, Namibia, Republic of the Congo, Senegal, South Africa, Tanzania, Zambia. **Australasia**–Australia (New South Wales, Queensland, Victoria), Hawaii, New Zealand, Tasmania. **Nearctic**–Canada (Alberta, British Columbia, Manitoba, Saskatchewan). USA (Colorado, Idaho, Illinois, Iowa, Kansas, Minnesota, Missouri, Montana, Nebraska, North Dakota, Oregon, South Dakota, Utah, Virginia, Washington, Wisconsin, Wyoming). **Neotropical**–Argentina (no further data), Mexico (no further data), Venezuela. **Oriental**–India. **Palaearctic**–Austria, Azerbaijan, Belgium, Bulgaria, China (Heilongjiang, Inner Mongolia, Qinghai, Shaanxi, Tibet, Xinjiang), Corsica, Czech Republic, Denmark, England, Finland, France, Germany, Hungary, Iran, Iraq, Italy, Kazakhstan,

Kyrgyzstan, Lithuania, Malta, Mongolia, Morocco, Palestine, Poland, Romania, Russia (Tomsk, Transbaikal, Yakutsk, Yenisseisk), Scotland, Slovakia, Sweden, Switzerland, Tajikistan, The Netherlands, Turkey, Turkmenistan, Ukraine, Uzbekistan.

***Gasterophilus inermis* (Brauer)**

Diagnosis. Facial plate bare. Wing partly infuscate, with ambiguously defined brownish spots. Distance between crossvein r-m and dm-cu obviously less than length of r-m. Meron bare. Legs yellowish brown, with femora distinctly darkened. Abdomen ground colour yellow. Male cercus short and broad, length-width ratio less than 1.0; surstylus yellow, with rounded apex. Egg yellowish, stalkless, elongate ovoid in shape, with the broad chorionic flanges accounting for 7/10 of the total egg length.

Distribution. **Afrotropical**–Senegal, South Africa. **Nearctic**–USA (Illinois). **Palaearctic**–Austria, China (Inner Mongolia, Xinjiang), France, Hungary, Iran, Italy, Kazakhstan, Kyrgyzstan, Moldavia, Mongolia, Slovakia Republic, Tajikistan, Turkmenistan, Ukraine, Uzbekistan.

Material examined. AUSTRIA: 1♀, 31.VII.1986, leg. Waldegg (NHMW); 1♂, 1892, no further data (NHMW); 1♂, 1♀, F. Brauer leg., no further data (NHMW); HUNGARY: 1♀, G. Dinulescu leg., no further data (MNHN); 1♂, 1918, 1♀, Wüstnei leg. (NHMD); 1♂, leg. G. Dinulescu, no further data (MNHN). CHINA: 1♂, Chifeng, Zhaowuda League, Right Banner, 16.VIII.1969, collector unknown (IOZ); 1♀, Chifeng, Zhaowuda League, Right Banner, 22.VIII.1969, collector unknown (IOZ).

References

1. Olivier GA. Encyclopédie méthodique. Histoire naturelle. Insectes. Paris: Histoire naturelle; 1811.
2. Brauer F. Monographie der Oestriden. Wien: W. Braumüller; 1863.
3. Zumpt F, Paterson HE. Studies on the family Gasterophilidae, with keys to the adults and maggots. J Entomol Soc South Afr. 1953;16: 59–72.
4. Patton WS. Studies on the higher Diptera of medical and veterinary importance. Ann Trop Med Parasitol. 1937;31: 351–359.
5. Grunin KJ. Gastrophilidae. In: E. Lindner, editor. Fauna URSS, Insecta: Diptera. Moscow: Education of Academy of Sciences of USSR; 1955. pp. 1–96.
6. Grunin KJ. Gasterophilidae. In: E. Lindner, editor. Die Fliegen der Paläarktischen Region. Stuttgart: Schweizerbart'sche; 1969. pp. 1–66.
